# Mitochondria-localized AMPK responds to local energetics and contributes to exercise and energetic stress-induced mitophagy

**DOI:** 10.1073/pnas.2025932118

**Published:** 2021-09-07

**Authors:** Joshua C. Drake, Rebecca J. Wilson, Rhianna C. Laker, Yuntian Guan, Hannah R. Spaulding, Anna S. Nichenko, Wenqing Shen, Huayu Shang, Maya V. Dorn, Kian Huang, Mei Zhang, Aloka B. Bandara, Matthew H. Brisendine, Jennifer A. Kashatus, Poonam R. Sharma, Alexander Young, Jitendra Gautam, Ruofan Cao, Horst Wallrabe, Paul A. Chang, Michael Wong, Eric M. Desjardins, Simon A. Hawley, George J. Christ, David F. Kashatus, Clint L. Miller, Matthew J. Wolf, Ammasi Periasamy, Gregory R. Steinberg, D. Grahame Hardie, Zhen Yan

**Affiliations:** ^a^Center for Skeletal Muscle Research at Robert M. Berne Cardiovascular Research Center, University of Virginia School of Medicine, Charlottesville, VA 22908;; ^b^Department of Human Nutrition, Foods, and Exercise, Virginia Polytechnic Institute and State University, Blacksburg, VA 24061;; ^c^Department of Biochemistry and Molecular Genetics, University of Virginia School of Medicine, Charlottesville, VA 22908;; ^d^Department of Pharmacology, University of Virginia School of Medicine, Charlottesville, VA 22908;; ^e^Department of Medicine, University of Virginia School of Medicine, Charlottesville, VA 22908;; ^f^Department of Microbiology, Immunology and Cancer Biology, University of Virginia School of Medicine, Charlottesville, VA 22908;; ^g^Department of Biomedical Engineering, University of Virginia School of Medicine, Charlottesville, VA 22908;; ^h^Department of Nephrology, University of Virginia School of Medicine, Charlottesville, VA 22908;; ^i^W. M. Keck Center for Cellular Imaging, University of Virginia, Charlottesville, VA 22904;; ^j^Department of Biology, University of Virginia, Charlottesville, VA 22904;; ^k^Department of Cardiothoracic Surgery, Stanford University School of Medicine, Stanford, CA 94305;; ^l^Centre for Metabolism, Obesity and Diabetes Research, Department of Medicine, McMaster University, Hamilton ON L8N 3Z5, Canada;; ^m^Department of Medicine, McMaster University, Hamilton ON L8N 3Z5, Canada;; ^n^Division of Cell Signaling and Immunology, School of Life Sciences, University of Dundee, DD1 5EH Scotland, United Kingdom;; ^o^Department of Orthopaedic Surgery, University of Virginia School of Medicine, Charlottesville, VA 22908;; ^p^Department of Public Health Sciences, University of Virginia School of Medicine, Charlottesville, VA 22908;; ^q^Center for Public Health Genomics, University of Virginia School of Medicine, Charlottesville, VA 22908;; ^r^Department of Biochemistry and Biomedical Sciences, McMaster University, Hamilton ON L8N 3Z5, Canada;; ^s^Department of Molecular Physiology and Biological Physics, University of Virginia School of Medicine, Charlottesville, VA 22908

**Keywords:** mitochondria, AMPK, skeletal muscle, exercise, mitophagy

## Abstract

Mitochondria form a complex, interconnected reticulum that is maintained through coordination among biogenesis, dynamic fission, and fusion and mitophagy, which are initiated in response to various cues to maintain energetic homeostasis. These cellular events, which make up mitochondrial quality control, act with remarkable spatial precision, but what governs such spatial specificity is poorly understood. Herein, we demonstrate that specific isoforms of the cellular bioenergetic sensor, 5′ AMP-activated protein kinase (AMPKα1/α2/β2/γ1), are localized on the outer mitochondrial membrane, referred to as mitoAMPK, in various tissues in mice and humans. Activation of mitoAMPK varies across the reticulum in response to energetic stress, and inhibition of mitoAMPK activity attenuates exercise-induced mitophagy in skeletal muscle in vivo. Discovery of a mitochondrial pool of AMPK and its local importance for mitochondrial quality control underscores the complexity of sensing cellular energetics in vivo that has implications for targeting mitochondrial energetics for disease treatment.

## Significance

Here, we present unequivocal evidence of physical association of AMPK holoenzymes with mitochondrial reticulum (mitoAMPK) across multiple mouse tissues with evidence of conservation in human skeletal muscle and heart. We demonstrate that mitoAMPK is activated heterogeneously across the mitochondrial reticulum by mitochondrial energetic stress. Finally, we present evidence that suggests activation of mitoAMPK in skeletal muscle is required for mitophagy. We propose that mitoAMPK responds to mitochondrial microenvironment cues to maintain energetic homeostasis through mitochondrial quality control.

Mitochondria form a complex, interconnected reticulum (1–4) that is maintained through orchestrated remodeling processes, such as biogenesis, dynamic fission and fusion, and targeted degradation of damaged/dysfunctional mitochondria, called mitophagy. These remodeling processes are collectively known as mitochondrial quality control and are initiated by various cues to maintain energetic homeostasis, which is particularly important for tissues with high-energy demands (e.g., skeletal muscle and heart) (5, 6). While the reticulum appears to respond to energetic demand uniformly (1, 2, 7), mitochondrial quality control acts with remarkable subcellular precision (8). For example, in both skeletal muscle and heart, impaired or damaged regions of mitochondria are separated from the functional reticulum in response to certain cellular signals, setting the stage for their degradation by mitophagy (1, 3, 9–13). However, what governs the spatial specificity of this process is poorly understood.

The cellular energy sensor, 5′-AMP-activated protein kinase (AMPK), is a heterotrimeric holoenzyme consisting of three subunits: a catalytic α (α1 or α2), a scaffolding β (β1 or β2), and a regulatory γ (γ1, γ2, or γ3) subunit (14). Canonically, AMPK senses cellular energy status by monitoring AMP and/or ADP levels. AMP and/or ADP bind to the γ subunit, resulting in a conformational change that exposes the T172 site of the catalytic α subunit to phosphorylation at T172 (15–19), fully activating AMPK (20). Muscle-specific knockout of both α subunit isoforms impairs exercises capacity and mitochondrial oxidative capacity (21), clearly linking energy sensing of AMPK to mitochondrial function as well as tissue function. Indeed, AMPK activation promotes mitochondrial fission in vitro through its direct substrate mitochondrial fission factor (Mff) (22). We and others have previously demonstrated that induction of mitophagy in response to energetic stress (e.g., exercise, fasting, etc.) is controlled by AMPK-dependent phosphorylation of Unc-51 like autophagy activating kinase 1 (Ulk1) at S555 in skeletal muscle (9, 23). In sum, AMPK integrates cell energetics to modulate mitochondrial quality control so to maintain energetic homeostasis.

To reconcile the subcellular specificity of mitochondrial quality control with the fact that exercise and other energetic stresses increase ADP and AMP (24, 25), the known activators of AMPK (26, 27), we hypothesized that a proportion and/or subtype of AMPK is localized at mitochondria. This pool of AMPK may serve as a gauge of energetic cues, particularly when and where ATP production through oxidative phosphorylation becomes limited. Herein, we uncovered that a particular combination of subunits of AMPK are localized to mitochondria in a variety of tissues, including skeletal muscle and heart in both mice and humans, which we term mitoAMPK. We show that mitoAMPK is localized to the outer mitochondrial membrane (OMM) and is activated in response to various stimuli of mitochondrial energetic stress. mitoAMPK activity and activation are spatially variable across the mitochondrial reticulum. Finally, we present evidence that suggests activation of mitoAMPK in skeletal muscle is required for mitophagy in vivo. Discovery of a pool of AMPK on mitochondria and its importance for mitochondrial quality control highlights the complexity of energetic monitoring in vivo and could facilitate development of strategies of targeting mitochondrial energetics to treat diseases related to impaired mitochondrial function.

## Results

### Identification of Enymatically Active AMPK on OMM.

**Fig. 1.**
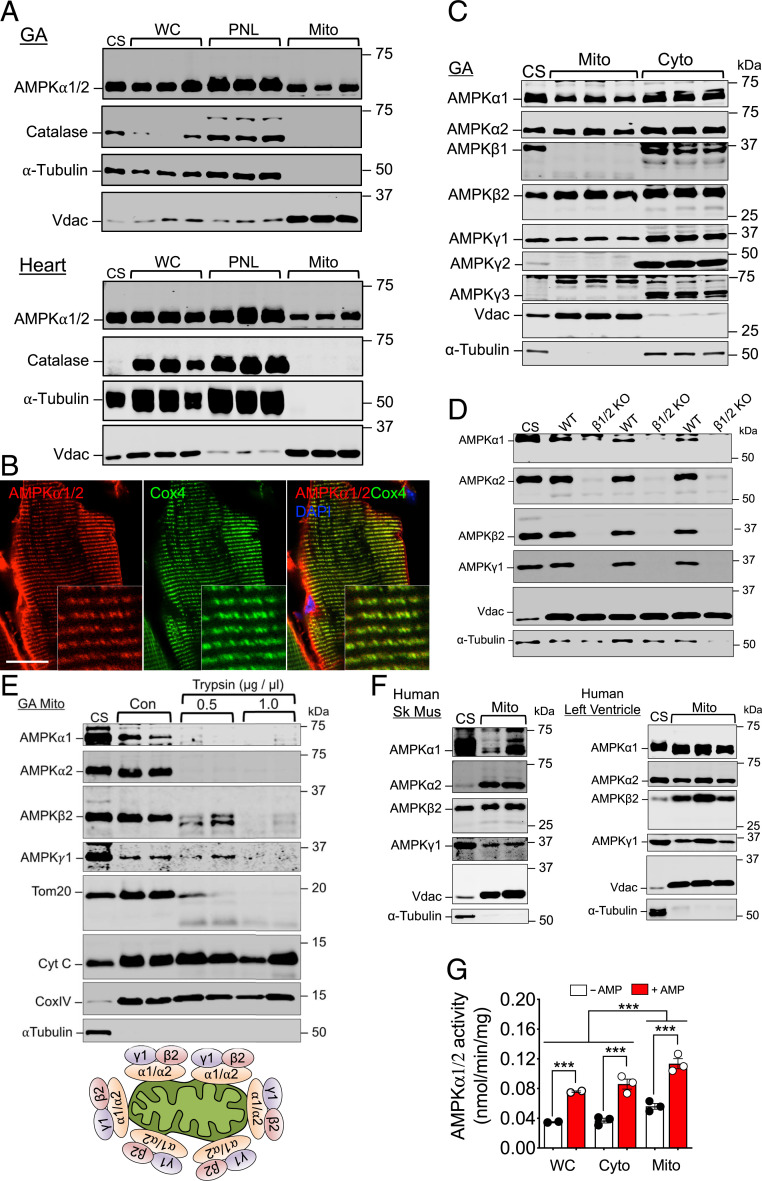
Identification of enymatically active AMPK on OMM in vivo. (*A*) Whole-cell lysates (WC), postnuclear lysates (PNL), and the corresponding enriched mitochondrial fractions of *Percoll* gradient isolation from mouse GA and Heart were probed for pan-AMPKα with Vdac, Catalase, and α-tubulin as loading and purity controls (*n* = 3). CS denotes mixed whole-tissue lysate comprised of mouse skeletal muscle, heart, and liver. (*B*) Immunofluorescence confocal microscopy of longitudinal sections of C57BL/6 mouse plantaris muscle probed by pan-AMPKα (red) and Cox4 (green) antibodies and DAPI for nuclear DNA (blue). Representative image of *n* = 3. (Scale bar, 20 µm.) (*C*) Enriched mitochondrial and cytosolic fractions isolated via differential centrifugation from mouse GA muscle and probed for each AMPK subunit isoform. *n* = 3. (*D*) Enriched mitochondrial fractions from frozen GA muscle of AMPKβ1/β2 knockout (KO) and wild-type littermate mice (WT). *n* = 3 per group. (*E*) Enriched mitochondrial fractions from mouse GA were treated with/without trypsin and probed for AMPKα1/α2/β2/γ1 (*n* = 2 per condition). An illustration of the physical association of AMPK with OMM is presented below. (*F*) Enriched mitochondrial fractions from human skeletal muscle biopsies (*n* = 2) and left ventricle biopsies (*n* = 3) were probed for AMPKα1/α2/β2/γ1. (*G*) AMPK activity in WC, cytosolic (Cyto), and enriched mitochondrial (Mito) factions with (+) and without (-) AMP (*n* = 3). All data presented as mean ± SEM. ***P < 0.001 by two-way ANOVA.

To determine whether AMPK is physically present in or associated with mitochondria in vivo, we isolated mitochondria from adult mouse gastrocnemius (GA) skeletal muscle and heart by Percoll gradient centrifugation (*SI Appendix*, Fig. S1*A*), a gold standard method of mitochondrial isolation for striated muscles (28), and performed immunoblotting using anti-AMPK pan-α antibodies. We readily detected AMPKα1/2 in these enriched mitochondrial fractions (Fig. 1*A*). We also detected AMPKα1/2 in isolated mitochondrial fractions via differential centrifugation from mouse GA and tibialis anterior (TA) skeletal muscles, heart, and kidney as well as liver (*SI Appendix*, Fig. S1*B*). Importantly, we confirmed that enriched mitochondrial fractions were free of cytosolic (evidenced via absence of α-Tubulin expression), endoplasmic reticulum (ER) (evidenced via absence of Sec61a expression) (29), or peroxisomes (evidenced via absence of Catalase expression) (Fig. 1*A* and *SI Appendix*, Fig. S1 *A* and *B*), suggesting that our detection of AMPK in enriched mitochondrial fractions was not due to contamination from other organelles. Also, confocal immunofluorescence microscopy of longitudinal sections of mouse plantaris skeletal muscle revealed a significant overlap of AMPK signal with mitochondrial electron transport chain protein, cytochrome oxidase 4 (Cox4) in the intermyofibrillar mitochondrial network (Fig. 1*B*).

Given the heterotrimeric structure of AMPK holoenzyme and the variation in possible subunit isoform composition across tissues (14), we used isoform-specific AMPK antibodies for detection of AMPK isoforms in enriched mitochondrial fractions from various tissues. We detected AMPKα1, α2, β2, and γ1 subunit isoforms in mouse GA and TA muscles and heart as well as differing variations of isoforms in kidney and liver (Fig. 1*C* and *SI Appendix*, Fig. S1*C*). The identities of α1, α2, β2, and γ1 isoforms were confirmed in enriched mitochondrial fractions of mouse GA by antigen peptide blocking, in which the primary antibodies were preincubated by antigen peptides for the respective antibodies (*SI Appendix*, Fig. S1*D*). We also performed cross blocking and demonstrated that the antigen peptides we used were specific for the designated antibodies (*SI Appendix*, Fig. S1*E*). Importantly, we took advantage of GA muscle of AMPKβ1/2 double-knockout mice, which has previously been shown to have significantly reduced expression of all AMPK subunits (30) and observed a complete loss of AMPK α1, α2, β2, and γ1 isoforms in enriched mitochondrial fractions isolated from frozen tissue (Fig. 1*D*). Furthermore, using CRISPR/Cas9-mediated gene editing, we generated AMPKα2(T172A) knock-in mice and confirmed loss of phosphorylation of AMPKα2 without a change of total AMPK in enriched mitochondrial fractions (*SI Appendix*, Fig. S1 *E* and *F*). Finally, to determine the precise location of AMPK isoforms in mitochondria, we performed a gradual trypsin digestion of isolated mitochondria from skeletal muscle and observed disappearance of α1, α2, β2, and γ1 subunits along with the OMM protein Tom20, while the inner mitochondrial membrane proteins Cox4 and Cytochrome C remained intact (Fig. 1*E*). Most importantly, we detected α1, α2, β2, and γ1 isoforms in enriched mitochondrial fractions from human skeletal muscle and heart (Fig. 1*F*), indicating that the physical association of AMPK with mitochondria is conserved in humans.

AMPK activation is a three-step process with allosteric binding of AMP or ADP to the γ subunit promoting enhanced net phosphorylation by upstream kinases and phosphatases of the catalytic α subunit at residue T172 (15–19), leading to full activation (20). We performed immunoprecipitated kinase assay (31) using enriched mitochondrial fractions isolated via Percoll gradient centrifugation and detected AMPK activity with clear evidence of allosteric activation by AMP (Fig. 1*G*). Using antibodies against catalytic α1 and α2 isoforms, we show the presence of AMPK activities for both isoforms with α2 being more active than α1 across different subcellular fractions (*SI Appendix*, Fig. S1*H*). The data presented in *SI Appendix*, Fig. S1 *E* and *F* for the CRISPR/Cas9-generated AMPKα2(T172A) knock-in mice are also consistent with the kinase assay data. We also performed bioinformatic analysis by mining three different protein–protein interaction (PPI) databases (32–34), which revealed 62 mitochondria-associated proteins (35 native mitochondrial proteins) that interact with AMPKα1 and 14 proteins (two native mitochondrial proteins) that interact with AMPKα2 (*SI Appendix*, Fig. S1*H* and Tables S1 and S2). Taken together, we present unequivocal evidence of physical association of enzymatically active AMPK of distinct isoforms with OMM, which we term mitoAMPK.

### mitoAMPK Is Activated by Mitochondrial Energetic Stress with Spatial Specificity.

**Fig. 2.**
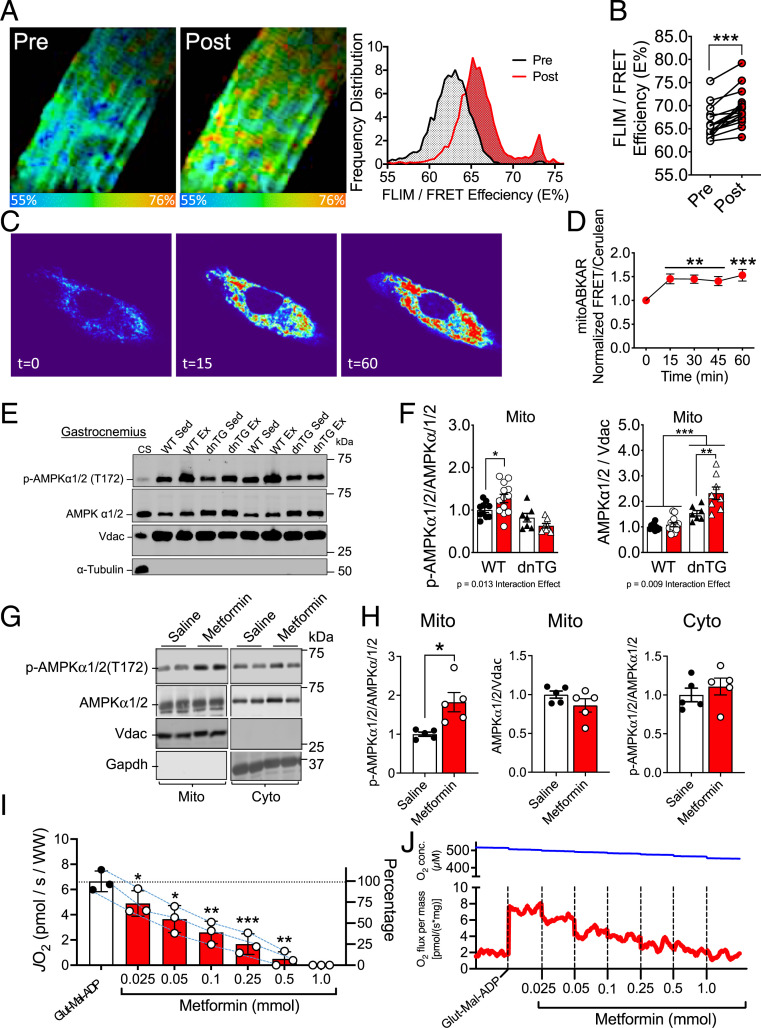
mitoAMPK is activated by mitochondrial energetic stress with spatial specificity. (*A*) Single FDB muscle fibers from C57BL/6 mice transfected with *pmitoABKAR* were cultured on phenol-red–free Matrigel coated glass plates, and FLIM/FRET efficiency (E%) was measured at rest and following 20 min of electrical stimulation-induced contractions and illustrated as representative heat map images (*Left*) and histogram (*Right*). (*B*) Calculated mean E%. *n* = 18 fibers across four independent experiments. (*C*) Representative heat map image of C2C12 myoblasts transfected with *pmitoABKAR* and imaged via confocal microscopy prior to 15 and 60 min following administration of 2.5 µM Oligomycin. (*D*) Data presented as normalized FRET ratio (FRET/cerulean). *n* = 21 cells per timepoint across two independent experiments. (*E*) Enriched mitochondrial fractions from GA muscles of sedentary (Sed) or immediately after 90 min of gradient treadmill running (Ex) in dnAMPKα2 and WT littermate mice were probed for p-AMPKα1/2 (T172) and pan-AMPKα. WT-sed (*n* = 13), WT-Ex (*n* = 13), dnTG-Sed (*n* = 7), and dnTG-Ex (*n* = 8). (*F*) Quantitative data of phosphorylated AMPK relative to total AMPK and total AMPK relative to Vdac. (*G*) Enriched mitochondrial fractions and corresponding cytosolic fractions from GA muscles of sedentary mice following 3 d metformin treatment (250 mg/kg via I.P.) or saline were probed for p-AMPKα1/2 (T172) and pan-AMPKα. For both groups, *n* = 5. (*H*) Quantitative data of phosphorylated AMPK relative to total AMPK in both Mito and Cyto fractions as well as total AMPK relative to Vdac. (*I*) Oxygen consumption rates of permeabilized TA muscle fibers in the presence of glutamate (10 mM) and malate (1 mM) were added to determine complex I leak respiration in the presence of physiological free ADP levels (20 µM) followed by titration of Metformin into the chamber (*n* = 3, run in triplicate). (*J*) Representative trace of complex I leak respiration during metformin titration. All data are presented as mean ± SEM. Results of the paired Student’s *t* test (*B*), one-way ANOVA (*D*), two-way ANOVA (*F*)**,** unpaired Student’s *t* test (*H*), and repeated measures ANOVA (*I*) are *P < 0.05, **P < 0.01, and ***P < 0.001.

Mitochondria present a reticular structure in most cells, including the heart and skeletal muscle, in which the reticulum extends across the entire length of myofibers (1, 2, 9, 10). To visualize mitoAMPK activity in adult skeletal muscle, we performed fluorescent lifetime Förster resonance energy transfer microscopy (FLIM/FRET) in cultured myofibers from flexor digitorum brevis (FDB) muscle transfected with the AMPK biosensor, mitoABKAR (*SI Appendix*, Fig. S2*A*) (35). mitoABKAR consists of an AMPK substrate sequence that is flanked by a cerulean donor and a venus variant acceptor that is targeted to OMM via a DAKAP1 targeting sequence (35). We digested and cultured single FDB fibers at least 10 d after transfection and measured FLIM/FRET energy transfer efficiency of the donor (E%) as an indicator of mitoAMPK activity prior to and immediately following 20 min of electrical stimulation-induced contractions (Fig. 2*A* and Movie S1). Muscle contractions resulted in increased mean E% (Fig. 2*B*) in a heterogeneous pattern (Fig. 2*A*). We also measured mitoAMPK activation by transfecting C2C12 myoblasts, which have endogenous mitoAMPK (*SI Appendix*, Fig. S2*B*), with *pmitoABKAR* and treating them with oligomycin to inhibit ATP synthase. As evidenced by an increase in acceptor fluorescence when only the cerulean donor was excited (i.e., FRET signal) relative to the donor fluorescence, oligomycin treatment significantly increased mitoAMPK activity with distinct regions of high FRET along the reticulum (Fig. 2 *C* and *D*), further illustrating that mitoAMPK is readily activated in a spatially distinct fashion as a result of mitochondrial energetic stress.

To investigate whether mitoAMPK is activated in response to energetic stress in vivo, we subjected C57BL/6J mice to various mitochondrial energetic stressors. When we subjected the heart and kidney to ischemia in mice via ligation of the left anterior descending artery (LAD) and renal vessel clamping, respectively, we obtained clear evidence of increased T172 phosphorylation in enriched mitochondrial fractions (*SI Appendix*, Fig. S2 *D*–*G*). Importantly, when we subjected mice to acute treadmill running (90 min), we detected increased T172 phosphorylation in enriched mitochondrial fractions from GA muscle immediately after exercise, but not in muscle-specific dominant-negative AMPKα2 transgenic littermate mice (dnTG) (Fig. 2 *E* and *F*). We also observed increased AMPK T172 phosphorylation in both cytosolic and enriched mitochondrial fractions following repeated contractions evoked by electrical stimulation compared to nonstimulated contralateral control muscle (*SI Appendix*, Fig. S2*C*), as well as 60 min of hindlimb ischemia by tourniquet application (11, 36) compared with the contralateral TA muscle (*SI Appendix*, Fig. S2 *H* and *I*). Finally, we tested the impact of administration of metformin, an effective anti-diabetes drug that has shown to selectively inhibit mitochondrial respiratory-chain complex 1 (37). Daily intraperitoneal (i.p.) injection for 3 consecutive d resulted in a significant increase in mitoAMPK T172 phosphorylation but not that of cytosolic AMPK (Fig. 2 *G* and *H*). In skinned skeletal muscle fibers, in the presence of Glutamate/Malate and physiological ADP (20 µM) (38), titration of Metformin impairs Complex I-mediated respiration ex vivo (Fig. 2 *I* and *J*) but not respiration capacity in muscle fibers from mice treated with metformin via i.p. injections in the absence of metformin during the mitochondrial respiration assay (*SI Appendix*, Fig. S2*J*). These findings suggest that metformin-induced mitoAMPK activation is due to energetic stress caused by direct inhibition of the mitochondrial respiratory activity. Together, these findings provide strong evidence that mitoAMPK can be activated by a variety of mitochondrial energetic stressors, which is consistent with a recent finding in mouse liver that a mitochondrial pool of AMPK is activated in response to extreme energetic stress (39). It is noteworthy that total mitoAMPK did not show any significant changes under all conditions tested (Fig. 2 *E*–*H* and *SI Appendix*, Fig. S2 *C*–*I*) except for dnTG with elevated total AMPK in the mitochondrial fraction due to transgenic overexpression (Fig. 2 *E* and *F*). In sum, these data suggest that mitoAMPK activation in vivo is not due to translocation of AMPK to mitochondria, as has recently been suggested in cell culture model systems (40).

### mitoAMPK Activity Regulates Mitochondrial Quality Control.

**Fig. 3.**
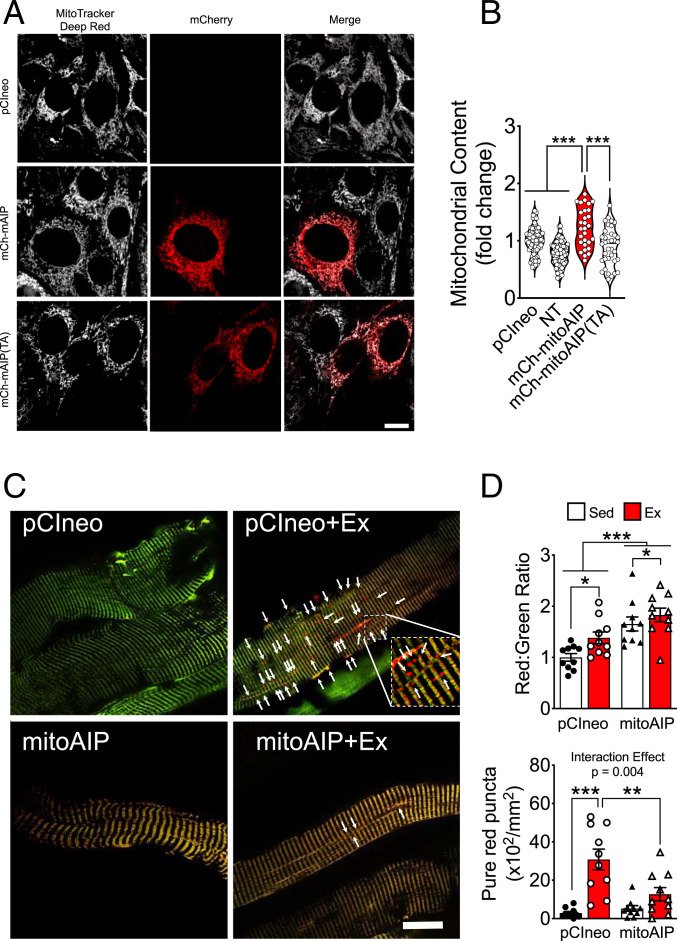
mitoAMPK activity regulates mitochondrial quality control. (*A*) Live confocal imaging of C2C12 myoblasts transfected with *pCIneo*, *pmitoAIP*, and *pmitoAIP(TA)* carrying mCherry (red) and stained with MitoTracker Deep Red (gray) with nontransfected cells (NT) as control. (*B*) Quantification of mitochondria occupied area as fold change relative to *pCI-neo* transfected cells. *pCIneo* (*n* = 73), NT (*n* = 78), *pmitoAIP* (*n* = 29), and *pmitoAIP(TA)* (*n* = 30) between three independent experiments. (*C*) Representative images of C57BL/6J mouse (10 to 12 wk) FDB fibers cotransfected with either *pMitoTimer* and *pCIneo* or *pMitoTimer* and *pmitoAIP(-mCherry)*. Images are merged red and green channels. (Scale bar, 20 µm.) (*D*) Quantification of MitoTimer Red:Green fluorescence intensity and pure red puncta. *n* = 10 per group. Data presented as mean ± SEM. Results of one-way (*B*) or two-way ANOVA (*D*) are *P < 0.05, **P < 0.01, and ***P < 0.001.

To investigate the functional role for mitoAMPK, we transfected C2C12 myoblasts with *pmitoAIP* (35), which encodes a targeted AMPK inhibitor peptide (AIP) that consists of an AMPK-substrate sequence linked to an OMM targeting sequence and an mCherry fluorophore for microscopic detection (*SI Appendix*, Fig. S3*A*). Previous studies have shown that AIP acts as a kinase sink by “out-competing” downstream substrates without disrupting native AMPK activation or affecting other potential AMPK pools (35). We performed live-cell imaging via confocal microscopy 24 h posttransfection following staining with MitoTracker Deep Red. Transfection with *pmitoAIP* led to significantly greater mitochondrial content compared with adjacent nontransfected cells or cells transfected with the empty vector, *pCIneo*, or *pmitoAIP(TA)*, which has the AMPK-targeted threonine mutated to alanine (Fig. 3 *A* and *B*). Increased mitochondrial content following inhibition of mitoAMPK activity is reminiscent of previous findings by Egan et al. in primary hepatocytes in which ablation of either AMPK or the mitophagy regulator Ulk1 led to accumulation of mitochondria due to impaired mitophagy (41). However, transfection of *pmitoAIP* or *pmitoAIP(TA)* did not prevent morphological changes related to mitochondrial fragmentation, induced by treatment with oligomycin and antimycin A (*SI Appendix*, Fig. S3 *C* and *D*) assessed by the MitoHacker imaging analysis platform (42). The findings suggest that under the extreme condition of oligomycin and antimycin (OA) treatment, inhibition of mitoAMPK is not sufficient to mitigate mitochondrial fragmentation. However, these experimental findings do not exclude the possibility that mitoAMPK may regulate mitochondrial dynamics, such as mitochondrial fission, under physiological conditions.

We have previously shown that endurance exercise induces mitophagy through AMPK-dependent phosphorylation of Unc-51 like Ulk1 at Ser555 (9). To determine whether mitoAMPK is required for mitophagy in vivo, we cotransfected mouse FDB muscle via electroporation (*SI Appendix*, Fig. S3*B*) with *pmitoAIP(-mCherry)*, which is *pmitoAIP* with in-frame removal of the mCherry sequence (*SI Appendix*, Fig. S3*A*), and *pMitoTimer*, a mitochondrial reporter for visualizing mitochondrial oxidative stress and mitophagy (3, 9, 10, 43, 44). MitoTimer fluoresces like green fluorescence protein (GFP; Ex/Em = 488/518 nm) when newly synthesized and irreversibly changes its fluorescence to that of Discosoma sp. red fluorescent protein (DsRed; Ex/Em = 543/572 nm) when oxidized (10). Cotransfection of the contralateral FDB with *pCIneo* and *pMitoTimer* were used as control. *pmitoAIP(-mCherry)* transfection resulted in a significant increase in the MitoTimer Red:Green ratio compared to *pCIneo* transfection in the contralateral FDB muscle (Fig. 3 *C* and *D*), indicating increased mitochondrial oxidative stress. The data suggest that inhibition of mitoAMPK leads to mitochondrial oxidative stress. We then subjected transfected mice to 90 min of treadmill running and performed confocal microscopy at 6 h post exercise, which corresponds to a peak of mitochondrial oxidative stress and mitophagy (9). Treadmill running resulted in a significant increase in MitoTimer Red:Green ratio in pCIneo-transfected FDB muscle compared with sedentary mice as previously observed (9), whereas the contralateral *pmitoAIP(-mCherry)*–transfected FDB muscle had a further increase from the elevated Red:Green ratio following exercise (Fig. 3 *C* and *D*). *pMitoTimer* pure red puncta are mitochondria containing autophagolysosomes (positive for both mitochondrial protein Cox4 and lysosomal marker Lamp1) (3, 9, 10). Here, we observed significantly increased pure red puncta in *pCIneo* transfected FDB following exercise, indicative of increased mitophagy (9) but not in the contralateral FDB cotransfected with *pmitoAIP(-mCherry)* (Fig. 3 *C* and *D*), suggesting an attenuation of exercise-induced mitophagy. Therefore, activation of mitoAMPK may be required for mitophagy induced by mitochondrial energetic stress, including endurance exercise.

## Discussion

From yeast to humans, AMPK has been credited with the control of diverse responses and adaptations to energetic challenges to maintain homeostasis. The notion that AMPK exists in distinct subcellular domains has only relatively recently been given consideration in the literature (45, 46), with the recent findings of Zong et al. being the first to show a mitochondrial pool of AMPK in vivo (39). Herein, we show that mitoAMPK in vivo consists of distinct isoforms with variation between different tissues (Fig. 1*C* and *SI Appendix*, Fig. S1*C*). We have obtained direct evidence that α1, α2, β2, and γ1 isoforms are localized to the OMM in skeletal muscle (Fig. 1*E*), and their association with mitochondria is at least conserved in human skeletal muscle and heart (Fig. 1*F*). We also show evidence for a localized activation of mitoAMPK (Fig. 2) and in governing mitochondrial quality control in response to energetic stress in skeletal muscle (Fig. 3), which may be related to the highly spatial activation (Fig. 2*A*). Future studies should elucidate the mechanism underlying mitoAMPK localization and other local signaling pathways that may govern different aspects of mitochondrial metabolism.

Recently, Zong et al. described a mitochondrial pool of AMPK in mouse liver that was activated in response to severe nutrient stress and ischemia (39); however, the specific isoforms that comprised this mitochondrial pool of AMPK were not elucidated. In the present study, we found physical presence of AMPK in enriched mitochondrial fractions from multiple tissues (Fig. 1*C* and *SI Appendix*, Fig. S1*C*). We confirmed in skeletal muscle that AMPK α1, α2, β2, and γ1 isoforms are localized to the OMM (Fig. 1*E*) and that their association with mitochondria is conserved in human heart and skeletal muscle (Fig. 1*G*) as has been proposed (47). We detected more mitoAMPK α2 activity in skeletal muscle than that of α1 (*SI Appendix*, Fig. S1*G*), which is consistent with the difference of expression levels in skeletal muscle as previously reported (48). Whereas liver mitochondrial fractions appeared to be more enriched for α2 over α1, and heart and kidney appeared to be more biased toward α1 (*SI Appendix*, Fig. S1*C*). Thus, it appears that a mitochondrial pool of AMPK exists in most tissues, but the isoform composition of mitoAMPK is tissue dependent. Our overall findings support the notion that subcellular pools of AMPK may have unique functional roles (39, 49).

The metabolic dissimilarity between tissues may relate to the relative expression of mitoAMPK isoform composition. Comparison of ADP accumulation following brief periods of ischemia across multiple tissues in rats demonstrates that heart and liver accumulate ADP similarly, whereas the kidney is much more sensitive to ischemia and accumulates twofold more ADP than the heart and liver at the same time point (50). However, despite similar accumulation in ADP between the heart and liver, only AMPK phosphorylation in the liver increases in response to short periods of ischemia, whereas AMPK phosphorylation in the heart does not change (50). In the present study, ischemic periods were optimized for each given tissue (60 min in skeletal muscle and heart and as short as 5 min in kidney) to observe increased mitoAMPK phosphorylation. Work in reconstituted systems has shown that the isoform make-up of AMPK complexes determines their sensitivity to energetic nucleotides (51). Therefore, our present findings highlight an unappreciated complexity of AMPK in vivo. Given that there are compounds that target specific AMPK isoforms to modulate kinase activity (52, 53), our finding of the mitochondrial-specific isoforms of mitoAMPK may provide an avenue for development of targeted therapeutics.

The presence of both α1 and α2 catalytic isoforms in enriched mitochondrial fractions from myofibers may allude to isoform-specific roles in modulating mitochondrial remodeling. AMPKα1 and α2 sequences are 84.4% similar in humans (83.5% in mice), and the sequences on either side of the T172 activating site are identical (20). However, the use of synthetic peptides in reconstituted systems has demonstrated differences in substrate specificity between α1 and α2 (54), suggesting isoform-specific substrates. Our rudimentary PPI analysis between AMPKα1 and α2 supports the notion of distinct, though possibility related roles for the respective isoforms. It will be important for future studies to elucidate these possibilities as isoform-specific functions of AMPK may carry significant implications for diseases associated with mitochondrial dysfunction.

We demonstrated that various physiological energetic stressors (e.g., repeated muscle contractions, ischemia, and treadmill running) resulted in increased phosphorylation of T172 of mitoAMPKα. Furthermore, a 3-d treatment with the widely prescribed anti-diabetes drug metformin preferentially increased phosphorylation of T172 of mitoAMPKα in skeletal muscle along with evidence of metformin-mediated inhibition of mitochondrial respiratory complex I (Fig. 2 *G*–*J*), suggesting a role for mitoAMPK in metformin’s action in skeletal muscle. While our physiological data suggest that mitoAMPK responds to mitochondrial energetic stress, recent studies have illuminated the spatial specificity by which energetic stress is managed across the mitochondrial reticulum in myofibers (1, 55). Using FLIM microscopy of an OMM-targeted AMPK biosensor in cultured FDB myofibers, we observed distinct areas of high FLIM E% (indicative of AMPK activity) following 20 min of electrical stimulation-induced contractions (Fig. 2*A*). This observation, when combined with a ∼10 to 12% variation in E% across the mitochondria in cultured muscle fibers at baseline, illustrates a more nuanced response of mitoAMPK to energetic stress, compared with the binary “on-off” change observed by immunoblotting for T172 phosphorylation. It is known that AMP and/or ADP activates, whereas ATP inhibits AMPK (26, 27, 56). Therefore, accumulation of AMP or ADP at energetically stressed portions of the mitochondrial reticulum may lead to localized activation of mitoAMPK, in line with AMPK activation upon mitochondrial insults and low-energy conditions (57). The observation of heterogenous activation of mitoAMPK herein supports this notion.

**Fig. 4.**
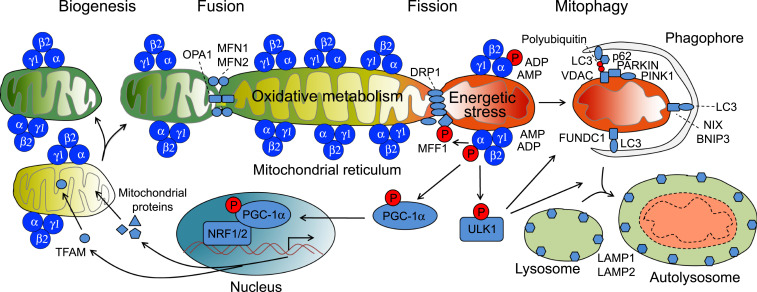
A working model of the regulation and function of mitoAMPK in mitochondrial remodeling. Mitochondrial energetic stress under the conditions of ischemia, muscle contraction, and/or pharmacological inhibition of mitochondrial respiratory chain will lead to subcellular increase of AMP and/or ADP in the vicinity of damaged/dysfunctional mitochondria (indicated by mitochondria of red color), which binds to and activates mitoAMPK (increased phosphorylation by upstream kinases). Activation of mitoAMPK promotes mitochondrial biogenesis, fission, and mitophagy through phosphorylating PGC-1a, MFF1, and ULK1, respectively. Cooperation of PGC-1a and NRF1/2 action in the nuclear genome with TFAM action in the mitochondrial genome drives mitochondrial biogenesis to add new, functional mitochondria (indicated by mitochondria of green color). Activated MFF1 interacts with DRP1 in executing mitochondrial fission for the physical separation the damaged/dysfunctional portion of mitochondria from the mitochondrial reticulum. Activated ULK1 promotes formation and targeting of autophagosome to damaged/dysfunctional mitochondria, which fuses with lysosome for degradation in autolysosome. This working model points to the central role of mitoAMPK in sensing mitochondrial energetic stress and regulating mitochondrial remodeling with subcellular specificity.

Seminal studies by Egan et al. demonstrate that genetic deletion of AMPK or Ulk1 in cultured primary hepatocytes results in an expansion of the mitochondrial reticulum, revealing the importance of this signaling pathway in mitochondrial clearance (41). We show here that competitive inhibition of mitoAMPK by transfection of an OMM-targeted AMPK substrate peptide (mitoAIP) but not the nonphosphorylatable peptide (mitoAIP(TA)) resulted in similar mitochondrial reticulum expansion in cultured myoblasts, suggesting a pivotal role of mitoAMPK in control of mitophagy. To reconcile the subcellular specificity of mitophagy (8, 9) with the fact that exercise and other energetic stresses increases ADP and AMP (24, 25), the known allosteric activator of AMPK (26, 27), we hypothesized that spatial variability in mitoAMPK activity and activation may implicate a role for mitoAMPK in sensing local mitochondrial energetics to modulate mitochondrial quality control. mitoAMPK may then serve as a gauge of mitochondrial energetic cues, particularly when and where ATP production through oxidative phosphorylation becomes limited. Using the established MitoTimer reporter gene for assessing mitophagy in vivo (3, 9–11, 36), we demonstrate that inhibition of mitoAMPK activity is sufficient to attenuate exercise-induced mitophagy in skeletal muscle. Based on these data, taken in context with spatial activation of AMPK across the reticulum in cultured myofibers, we propose a working hypothesis that mitoAMPK acts as an energetic surveillance mechanism to fine-tune mitochondrial remodeling, such as mitophagy, for maintenance of energetic homeostasis (Fig. 4). This notion is in line with the PPI model herein and expands upon known functions of AMPK in mitophagy (9), fission (22), and protein scaffolding (45). In the future, it will be important to discern how this local aspect of mitoAMPK in mitochondrial quality control coordinates with other known localized regulators of mitochondria, which may not be involved in the acute exercise response (58, 59).

In conclusion, we have demonstrated that a mitochondrial pool of AMPK is present in multiple tissues and consists of distinct isoforms that are conserved between mice and humans. Our data suggests that mitoAMPK is responsive to the energetic microenvironment of the mitochondrial reticulum in a way that modulates mitochondrial quality control mechanisms. Importantly, mitoAMPK activation is involved in mitochondrial quality control at least through the regulation of mitophagy. Collectively, these findings underscore the complexity by which energetic monitoring occurs in vivo. Elucidating the role of mitoAMPK in other pathways and how specific pathologies may interfere with its function on the mitochondria will be important areas of investigation going forward.

## Materials and Methods

### Animals.

All animal procedures were approved by the University of Virginia and Virginia Polytechnic Institute and State University Institutional Animal Care and Use Committees. All mice were housed in temperature-controlled (21 °C) quarters with 12:12-h light–dark cycle and ad libitum access to water and chow (Purina). Wild-type mice (C57BL/6J, male, 8 to 12 wk old) were obtained commercially (Jackson Laboratories) for mitochondrial isolation, ischemia, exercise, electrical-stimulated muscle contraction, and somatic gene transfer experiments. Male dnAMPKα2 mice and their wild-type littermates (13 to 15 wk old) were from colonies bred in house (9). AMPKα2(T172A) knock-in mice were generated using CRISPR/Cas9-mediated gene editing at Genetically Engineered Murine Model Core at University of Virginia as described (60). The target sequence for the guide RNA (gRNA) is 5′-GAA​TTT​CTA​CGA​ACT​AGC​TGG-3′, in which TGG is the protospacer adjacent motif (PAM) sequence and ACT is the codon for threonine 172 that is mutated to GCT for alanine.

### Cell Culture.

For determination of the impact of inhibition of mitoAMPK on mitochondrial fission, C2C12 myoblasts were cultured in 35-mm dishes and transfected at 30 to 40% confluency with *pmitoAIP* (0.5 μg) or the control plasmid *pmitoAIP(TA)* (0.5 μg) with Lipofectamine 2000 as described (61) for 24 h followed by treatment with oligomycin (10 μM) and antimycin (4 μM) for 3 h and stained with MitoTracker (0.5 μM) for 30 min and with DAPI (3.575 μM) for 10 min before fixation with 4% paraformaldehyde for epifluorescence microscopy.

### Bioinformatics.

Mouse and human amino acid sequences for AMPKα1, AMPKα2, AMPKβ1, AMPKβ2, AMPKγ1, AMPKγ2, AMPKγ3, and NDUFV2 were obtained from UniProt (62). A hypothesis generating AMPKα1 and AMPKα2 mitochondrial interactome was created by compiling the results for mitochondrial proteins between three open-source PPI databases BioGrid3.5 (32), GeneMania (33), and IntAct (34). Mitochondrial proteins were grouped according to their biological function(s) based on gene ontology and UniProt annotation.

### Human Tissue Procurement.

De-identified human muscle tissues were collected from the discarded tissues after standard surgical procedures (e.g., knee or shoulder reconstruction). Tissues were collected from the Department of Surgery at the University of Virginia School of Medicine. Institutional Review Board (IRB) approval was not required since samples had been de-identified. Human left ventricle biopsies were obtained at Stanford University from nondiseased donor hearts rejected for orthotopic heart transplantation and procured for research studies. Hearts were arrested in cardioplegic solution and transported on ice following the same protocol as hearts for transplant. The left ventricular free wall myocardium was dissected from epicardial adipose on ice, rinsed in cold phosphate-buffered saline, and rapidly snap-frozen in liquid nitrogen. Tissues were de-identified, and clinical information was used to select nonischemic hearts with left ventricular ejection fraction (LVEF) greater than 50%. Frozen tissues were transferred to the University of Virginia through a material transfer agreement and IRB approved protocols.

### Mitochondrial Fraction Isolation.

Mitochondrial-enriched lysates were isolated via differential centrifugation (9), or Percoll gradient fractionation from fresh GA and TA muscles, heart, liver, and kidney as described (63). For Percoll gradient isolation, whole-tissue lysates in fractionation (FRAC) buffer (20 mM Hepes, 250 mM Sucrose, 0.1 mM EDTA plus protease [Roche Diagnostics], and phosphatase [Sigma] inhibitors), were spun at 800 g for 10 min at 4 °C, and the resulting supernatant, termed postnuclear lysate, was layered on top of a 20 to 60% Percoll gradient and spun at 36,000 × g at 4 °C for 60 min. Mitochondrial layer was evident at the top of 20% Percoll layer, which was isolated and diluted in FRAC buffer and spun at 17,000 × g at 4 °C for 10 min. The pellet was resuspended in FRAC buffer and spun again until a solid pellet formed at bottom of the tube, designated as mitochondrial fraction (Mito). The whole-tissue lysates, postnuclear lysates, and mitochondrial fractions were each resuspended in Laemmli buffer containing phosphatase (Sigma) and protease (Roche Diagnostics) inhibitors and boiled for 5 min at 97 °C, then frozen at −80 °C until further analysis. For isolation of mitochondria-enriched fraction from C2C12 myoblasts, cells were scraped from plates in FRAC buffer containing protease (Roche Diagnostics) and phosphatase inhibitors (Sigma) and lysed by passing through a 20-gauge syringe at least 10 times. The mitochondria enriched fraction was isolated via differential centrifugation (9).

### Western Blotting.

Western blotting was performed as described previously (9) using the following primary antibodies: pan-AMPKα (CST no. 2532), p-AMPKα1/2(T172) (CST no. 2535), AMPKα1 (Abcam no. 3759), AMPKα1 antigen (Abcam no. 40461), AMPKα2 (Abcam no. 3760; NBP2-38532; NBP2-38532PEP), AMPKβ1 (CST no. 4178), AMPKβ2 (Novus Biologicals no. 92286), AMPKβ2 antigen (Novus Biologicals no. 92286PEP), AMPKγ1 (Abcam no. 32508), AMPKγ1 antigen (Abcam no. 218345), AMPKγ2 (Novus Biologicals no. 89324), AMPKγ2 antigen (Novus Biologicals no. 89324PEP), AMPKγ3 (Abcam no. 38228), Vdac (CST no. 4661), Tom20 (CST no. 42406), Cox4 (CST no. 4859), Gapdh (CST no. 2118), α-Tubulin (mouse, Abcam no. 7291), Catalase (rabbit, Abcam no. 16731), and Sec61a (CST no. 14867). Proteins were analyzed in comparison to a common protein standard loaded on the gel, which consisted of a whole-tissue lysate mixture of liver, heart, and skeletal muscle.

### Immunohistochemistry.

Plantaris muscle was harvested, prepared, and used for immunofluorescence as described (64) using anti-Cox4 (CST no. 2535) and rabbit pan-AMPKα (Abcam no. 131512) with appropriate negative control. Images were collected via confocal microscopy using Olympus Fluoview FV1000.

### Antigen Blocking.

Primary AMPKα1, AMPKα2, AMPKβ2, and AMPKγ1 antibodies were preincubated with/without 5× or 10× (α2 only) molar concentration respective antigen for 1 h at room temperature before used for Western blotting.

### Mitochondrial OMM Digestion.

Mitochondria fractions were incubated in 0.5 or 1.0 µg/µL Trypsin (Thermo) in FRAC buffer on ice for 15 min (65) followed by addition of 2 mM phenylmethylsulphonyl fluoride (Sigma) and incubation on ice for another 5 min. The lysates were then spun at 11,000 × *g* for 10 min at 4 °C, and the pellets were resuspended in Laemmli buffer plus protease (Roche Diagnostics) and phosphatase (Sigma) inhibitors and boiled for 5 min at 97 °C, then frozen at −80 °C until further analysis.

### AMPK Kinase Assay.

AMPK from cytosolic and mitochondrial fractions purified via Percoll gradient isolation was immunoprecipitated using anti-AMPKα1 (Abcam no. 3759), anti-AMPKα2 (Abcam no. 3760), and anti–pan-AMPKα (CST no. 2532) antibody and assayed as described previously (31).

### Acute Treadmill Running Exercise.

Acute treadmill running and prior acclimatization was performed as previously described (9).

### Hindlimb Ischemia.

Acute hindlimb ischemia was performed as previously described (11, 36).

### Cardiac Ischemia.

Cardiac ischemia was induced in vivo by ligation of the LAD artery (66, 67).

### Kidney Ischemia.

Kidney ischemia was induced by unilateral clamping of renal vessels (12975473). The nonischemic contralateral kidney was excised to serve as control.

### Electrical Stimulation.

Stimulation of the TA muscle was performed as previously described (11, 68) at 100-Hz stimulation frequency, 300-ms stimulation duration every second, 0.3-ms pulse duration, and 15-V electric potential for 20 min. Stimulation of cultured, single FDB muscle fibers was performed by insertion of a custom, three-dimensional (3D)–printed insert containing two parallel platinum electrodes ∼10 mm apart into a Attofluor cell chamber (Invitrogen) that was filled with Tyrode’s buffer (137 mM NaCl, 2.7 mM KCl, 1.8 mM CaCl_2_2H_2_O, 1 mM MgCl_2_6H_2_O, 0.2 mM NaH_2_PO_4_, 12 mM NaHCO_3_, and 5.5 mM Glucose, pH = 6.5). The 3D-printed stimulation insert has a rectangular opening in the center for microscope visualization. Stimulation was modified from previous studies (69–71), which consisted of 70-Hz stimulation frequency, 350-ms stimulation duration every 2 s, 0.3-ms pulse duration, and 150-V electric potential for 20 min.

### Metformin Treatment.

We performed daily intraperitoneal injection of metformin at 250 mg/kg for 3 d (72). GA muscles were harvested 1 h after the last injection.

### Mitochondrial Respiration.

Mitochondrial respiration was assessed via oxygen consumption rates of permeabilized muscle fibers measured through a high-resolution respirometry device (O2k, Oroboros Instruments, Innsbruck, Austria) and performed in triplicate. Methods for dissection, permeabilization, and specific substrate-uncoupler-inhibitor titration (SUIT) protocol for determining Complex I respiration were adapted from previously published protocols (73, 74). Briefly, one TA muscle from each mouse was dissected into fiber bundles ranges in size from 5 to 15 myofibers per bundle and permeabilized with 100 µg/µL of saponin in Buffer ×. Fiber bundles were then rinsed for 15 min in Buffer Z before being separated, and 2.5-mg portions were loaded into respirometry chambers. Measurements were performed in triplicates at 25 °C with constant stirring and oxygen concentration maintained between 500 and 300 µM/L. Baseline respiration rate was recorded after the fiber bundles were given time to equilibrate to the O2k chamber and before the addition of any respiration substrates. For metformin titration experiment, glutamate (10 mM), malate (1 mM), and ADP (20 µM) were added to determine complex I leak respiration in the presence of physiological free ADP levels in mouse skeletal muscle (38). Metformin was then titrated into the chamber at 0.025, 0.05, 0.1, 0.25, 0.5, and 1.0 mmol. Rates were allowed to stabilize for at least 4 min between additions. For postmetformin treatment experiments, the following was performed: First, glutamate (10 mM) and malate (1 mM) were added to determine complex I leak respiration, followed by succinate (10 mM) to determine combined complex I&II leak respiration. ADP (2.5 mM) was added at a saturating concentration to determine max state III respiration followed by the addition of rotenone (0.5 µM) to inhibit complex I. Complex I inhibition allowed for the determination of complex I contribution to state III respiration by calculating the percent decline of the state III respiration rate after the addition of rotenone which results in the percent of Complex I contribution to state III respiration. Cytochrome C (10 µM) was then added to assess mitochondrial membrane integrity, and any test in which the rate increased 10% after the addition of cytochrome C was excluded from analysis. Lastly, uncoupled respiration (maximum capacity) was achieved by the addition of carbonyl cyanide p*-*trifluoromethoxyphenylhydrazone (FCCP) (0.15 µM). Oxygen consumption rates were normalized to wet weight of tissue loaded into the chamber. Initial baseline respiration rates were subtracted from all rates before analysis.

### Plasmid DNA and Transfection.

Plasmid constructs were transfected into the FDB muscle by somatic gene transfer as previously described (9–11, 75).

### Culture of Single FDB Fibers.

FDB muscles were removed intact and placed in 1 mL collagenase solution (0.2% Type-II collagenase and 0.2% bovine serum albumin in Tyrode’s buffer with 1% PenStrep) in a 24-well culture plate. Muscles were incubated for 2 h. at 37 °C in a tissue culture incubator and agitated every 30 min. Muscles were then transferred to warmed 10% fetal bovine serum (FBS) Dulbecco's modified eagle medium (DMEM). Single muscle fibers were dispersed by passing gently through wide-mouthed plastic Pasteur pipette 30×. Aliquots of media containing single FDB fibers were placed on glass slides coated with Phenol-Red Free Matrigel and incubated at 37 °C in a tissue culture incubator for 30 min to adhere to the Matrigel. Phenol-Red Free DMEM with 10% FBS was then added to each slide for at least 30 min before any imaging was performed.

### FLIM/FRET Imaging.

FLIM/FRET is ideal for live-cell imaging, because the signal is independent of biosensor concentration and change in fluorescent excitation intensity but sensitive to changes in cellular environment, thus allowing measurement of dynamic events at very high temporal (nanoseconds) and spatial resolution (76). FLIM of cultured single FDB fibers before and after electrical stimulation was performed on a Zeiss 780 confocal/FLIM laser scanning microscopy controlled with Zen software (Carl Zeiss, Inc). Multiphoton excitation of the Cerulean donor of *pmitoABKAR* (35) was achieved by using a Ti:sapphire laser (Ex: 820 nm), operating at 80 MHz repetition rate (Chameleon Vision II, Coherent, Inc.). The fluorescence decay per pixel was measured using Time-Correlated Single Photon Counting (Becker & Hickl) in which single-photon counting module (SPCM) software was used to acquire the FLIM data (version 8.91). E% was calculated in SPCM software as E%2 = 1 – (τ1/τ2), with τ1 being the quenched donor lifetime and τ2 is the unquenched donor (77). Details of the FLIM set-up have been discussed elsewhere (78). A Zeiss 40× 1.3 NA oil (Zeiss EC Plan-Neofluar,) objective lens was used to focus the light on the sample and collect the emission for 16 s. The average power at the specimen plane (7 mW) and the acquisition time were chosen to reduce any photodamage to the cells. After acquisition of FLIM images for Cerulean, the fluorescent lifetime images were fitted for two components using SPCImage software (version 5.5, Becker & Hickl). FLIM efficiency (E%), donor lifetime, and photon images for each pixel was generated. For FRET imaging of C2C12 cells transfected with *pmitoABKAR*, cells grown on glass plates were transferred to live-cell imaging chambers (Invitrogen) in phenol-red–free DMEM with 20% FBS. Images were acquired at with Leica HCX PL APO CS 63x 1.4NA Oil ultraviolet (UV) lens at a resolution of 512 × 512 and 12 bits gray level and 1 airy unit on a Leica SP5 × confocal microscope. Cerulean donor was excited at 457 nm, and the FRET emission was captured at 475 to 503 nm. All images were acquired using identical parameters to ensure no signal saturation and similar intensity. Once the positively transfected cell had been identified and baseline images acquired, media was removed and replaced with serum and phenol-red free DMEM containing 2.5 µM Oligomycin. Subsequent images were acquired at 15, 30, 45, and 60 min after media change. Normalized FRET to Donor (Cerulean) ratio was calculated using the PFRET plugin in Image J (79).

### Confocal Microscopy.

MitoTimer in adult muscle fibers was performed as previously published (3, 9–11). For C2C12 cells transfected with *pmitoAIP*, cells on glass plates were stained with 400 nM MitoTracker Deep Red in DMSO (Sigma) for 30 min and transferred to live cell imaging chambers (Invitrogen) in phenol-red–free DMEM with 20% FBS. Images were acquired at 100× magnification (Olympus Fluoview FV1000) using red (via 543-nm laser) and far red (via 635-nm laser) channels through a tetramethylrhodamine (TRITC) (Ex/Em 555/580 nm) and Alexa Fluor 647 filters (Ex/Em 649/666 nm), respectively. Mitochondrial content as a percentage of cell volume was analyzed via mitochondria morphology macro in Image J (80).

### Statistical Analyses.

Data are presented as the mean ± SEM. Time course experiments are analyzed via one-way ANOVA with Newman–Keuls post hoc analysis. Where two variables are present, data were analyzed using two-way ANOVA with Tukey post hoc analysis where appropriate. Where one variable is present, data were analyzed using Student’s *t* test. Data with significantly unequal variance was transformed prior to statistical analysis. Statistical significance was established a priori as *P* < 0.05.

## Supplementary Material

Supplementary File

Supplementary File

## Data Availability

All study data are included in the article and/or supporting information.
