# Supplementary material for: Mitochondria-localized AMPK responds to local energetics and contributes to exercise and energetic stress-induced mitophagy
Source: Proc Natl Acad Sci U S A. 2021 Sep 7;118(37):e2025932118. doi: 10.1073/pnas.2025932118 (PMC8449344; doi:10.1073/pnas.2025932118)
Supplement: Supplementary File [file pnas.2025932118.sapp.pdf]

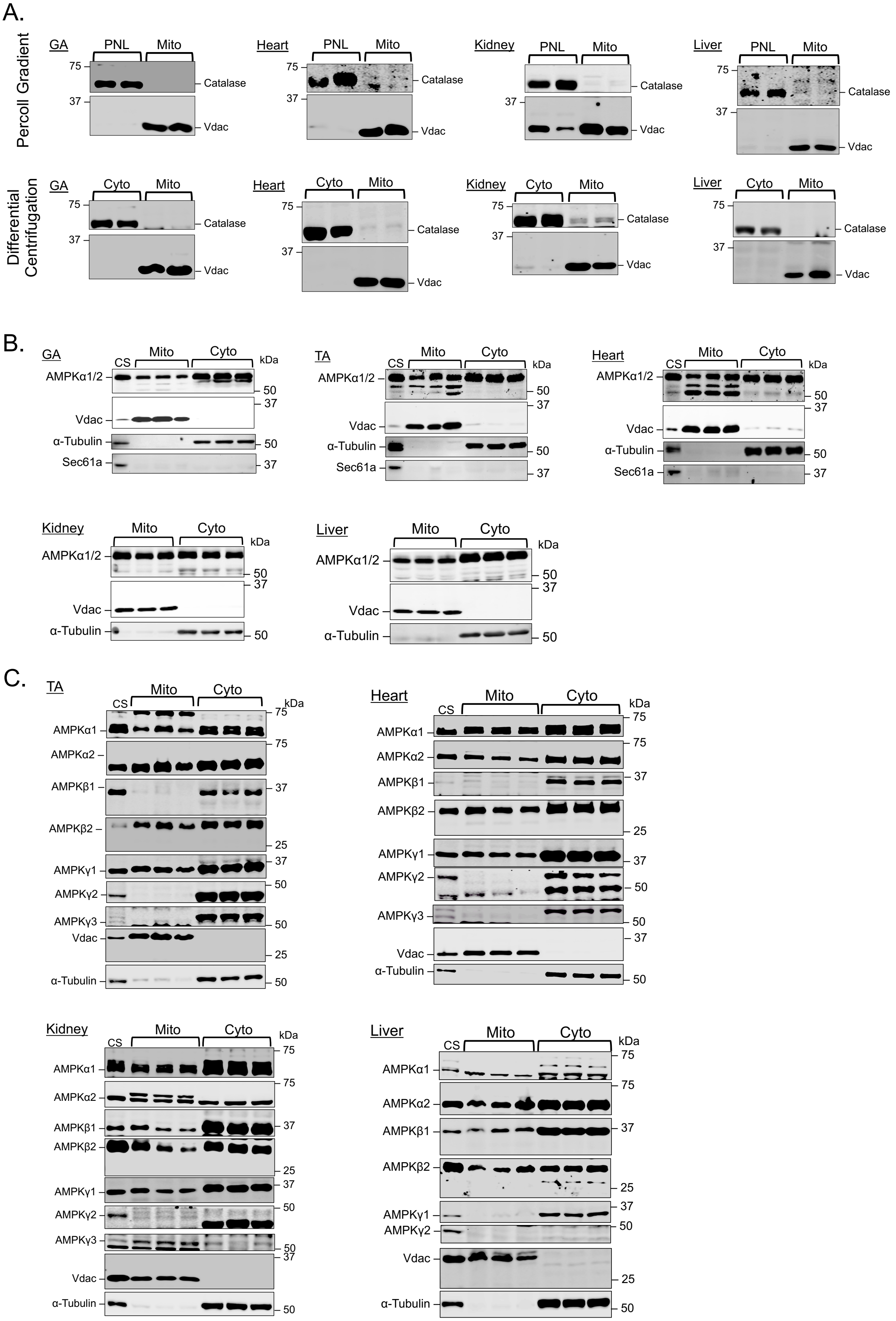
A.
Percoll Gradient
GA
PNL
Mito
Heart
Kidney
Liver
Catalase
Vdac
75
37
Differential Centrifugation
Cyto
B.
CS
kDa
AMPKα1/2
α-Tubulin
Sec61a
TA
50
C.
AMPKα1
AMPKα2
AMPKβ1
AMPKβ2
AMPKγ1
AMPKγ2
AMPKγ3
25

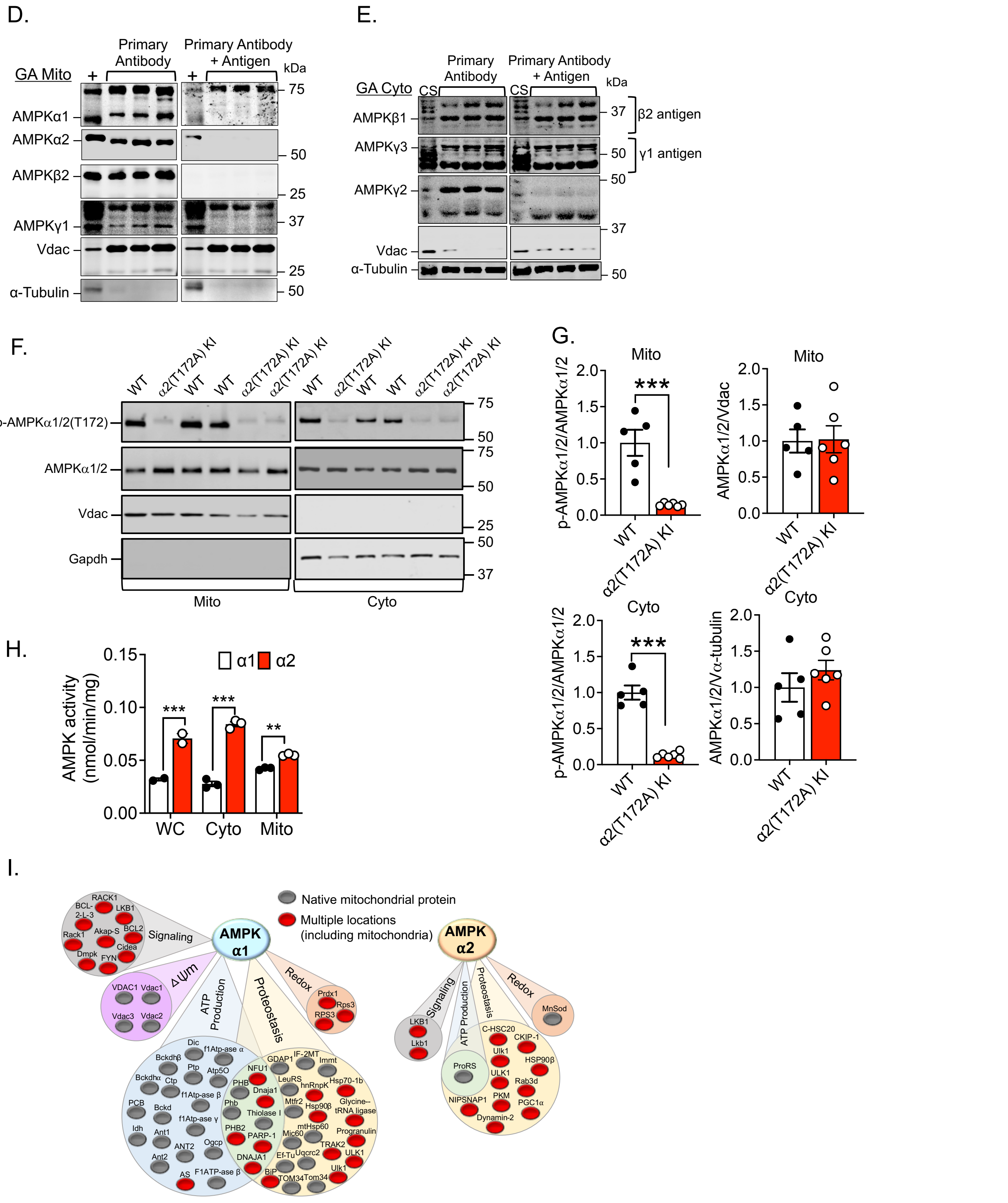

Figure S1. A. Verification of peroxisome (Catalase) contamination in enriched mitochondrial fractions isolated either by differential centrifugation or Percoll gradient. **B.** Enriched mitochondrial (Mito) and cytosolic fractions (Cyto) from mouse GA and TA muscle, heart, kidney and liver were probed for pan-AMPKα with Vdac, α-tubulin and Sec61a as loading and purity controls (n = 3). CS denotes mixed whole tissue lysates of mouse skeletal

muscle, heart and liver; **C**. Enriched mitochondrial (Mito) and cytosolic fractions (Cyto) from mouse TA muscle, heart, kidney and liver were probed for AMPK subunit isoforms (n = 3); **D**. Enriched mitochondrial fractions from GA muscle were probed for AMPKα1, α2, β2 or γ1 antibodies (left) or pre-incubated with 5x or 10x (α2 only) molar concentration of antibody-specific antigen (right) (n = 3); **E**. Enriched mitochondrial fractions from GA muscle were probed for AMPKβ1, γ3 or γ2 antibodies (left) or pre-incubated with 5x molar concentration of antibody-specific antigen (listed on the right except for no label for γ2 peptide) (n = 3); **F**. Enriched mitochondrial fractions from GA muscle of AMPKα2T172A knock-in (KI) and wild-type littermate mice (WT). WT (n = 5) and *AMPKα2T172A* KI (n = 6); **G.** Quantification of phosphorylated AMPK relative to total AMPK and total AMPK relative to Vdac for mitochondrial fraction (Mito) and phosphorylated AMPK relative to total AMPK and total AMPK relative to α-tubulin for cytosolic fraction (Cyto); **H**. AMPKα1 and α2 kinase assay following isoform-specific immunoprecipitation in whole muscle cell lysate (WC), cytosolic (Cyto) and enriched mitochondrial fractions (Mito) from mouse hindlimb skeletal muscle via Percoll gradient (n = 3); and **I**. Protein-protein interaction database search results for interaction between AMPKα1 and α2 and mitochondrial proteins. All data are presented as mean ± standard error of the mean. Results of the unpaired student's t-test (**F)** and two-way ANOVA (**G**) are ** $p < 0.01$ and *** $p < 0.001$.

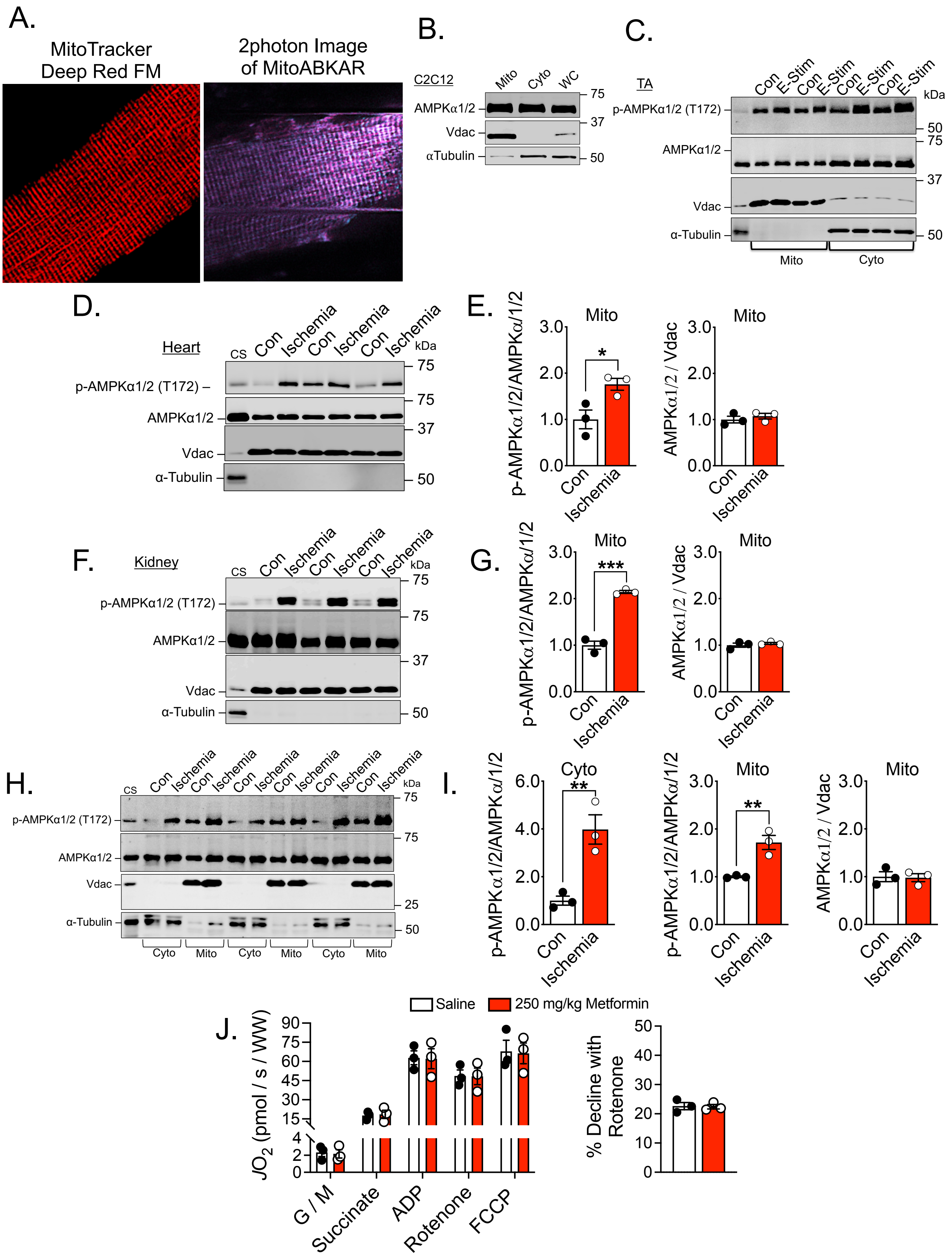
A.
MitoTracker Deep Red FM
2photon Image of MitoABKAR
B.
C2C12
Mito
Cyto
WC
AMPKα1/2
Vdac
αTubulin
75
37
50
C.
TA
Con
E-Stim
kDa
p-AMPKα1/2 (T172)
AMPKα1/2
Vdac
α-Tubulin
Mito
Cyto
D.
Heart
CS
Con
Ischemia
kDa
p-AMPKα1/2 (T172)
AMPKα1/2
Vdac
α-Tubulin
E.
Mito
p-AMPKα1/2/AMPKα1/2
AMPKα1/2 / Vdac
Con
Ischemia
F.
Kidney
G.
H.
Cyto
Mito
I.
Cyto
J.
Saline
250 mg/kg Metformin
JO_2 (pmol / s / WW)
G / M
Succinate
ADP
Rotenone
FCCP
% Decline with Rotenone

Figure S2. A. Comparison of cultured single mouse FDB fibers stained with MitoTracker^{TM} Deep Red and two-photon image of FDB fiber transfected with *pmitoABKAR*; **B**. Enriched mitochondrial (M) and cytosolic fractions (C), and whole cell lysates (WC) from cultured C2C12 myoblasts were probed for pan-AMPKα; **C**. Enriched mitochondrial and cytosolic fractions from mouse TA muscle harvested immediately after 20 min of direct muscle stimulation (Stim) using the contralateral non-stimulated TA muscle as control (Con) were p-AMPKα1/2 (T172) and pan-AMPKα with Vdac and α-tubulin as loading and purity controls (n = 2); **D**. Enriched mitochondrial fractions from sham control (Con) or mouse heart immediately following 60 min ischemia induced by LAD ligation (Ischemia) were probed for p-AMPKα1/2 (T172), pan-AMPKα with Vdac and α-tubulin as loading and purity controls. n = 3; **E**. Quantitative data of phosphorylated AMPK relative to total AMPK and total AMPK relative to Vdac; **F,** Enriched mitochondrial fractions from mouse kidney harvested immediately after 5 min kidney ischemia via clamping of the kidney vessels (Ischemia) were probed for p-AMPKα1/2 (T172) and pan-AMPKα and compared to fractions from contralateral non-ischemic kidney (Con). n = 3. **G**. Quantitative data of phosphorylated AMPK relative to total AMPK and total AMPK relative to Vdac; **H**. Enriched mitochondrial and cytosolic fractions from mouse TA muscle harvested immediately after 60 min of hindlimb ischemia via tourniquet without reperfusion were probed for p-AMPKα1/2 (T172) and pan-AMPKα with Vdac and α-tubulin as loading and purity controls and compared to fractions from contralateral, non-ischemic limb (n = 3). **I**. Quantification of phosphorylated AMPK relative to total AMPK and total AMPK relative to Vdac for mitochondrial fraction (Mito); **J.** Mitochondrial oxygen consumption normalized to mg wet weight of permeabilized muscle fibers after the addition of glutamate/malate, succinate, ADP, rotenone, and FCCP following three days of metformin administration (i.p.). Also, Quantification of the percent decline after the addition of rotenone is shown to represent Complex I contribution to State III respiration. All data are presented as mean ± standard error of the mean. Results of unpaired student's t-tests (E, G, I, and J) are * $p < 0.05$, ** $p < 0.01$, and *** $p < 0.001$.

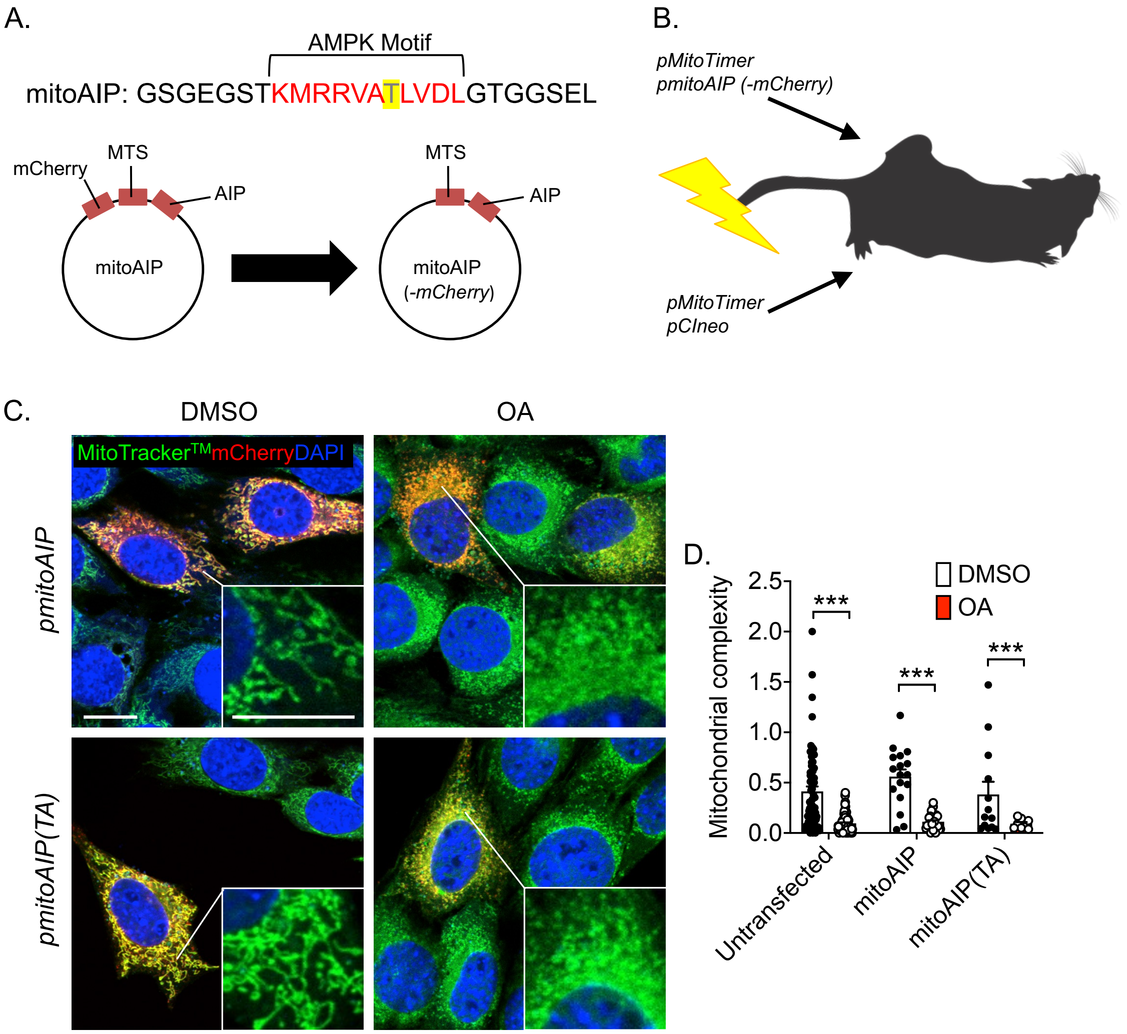

Figure S3. mitoAMPK activity regulates mitochondrial quality control. A. DNA structure of *pmitoAIP* and *pmitoAIP(-mCherry)* used in studies presented in Fig. 3A-B and 3C-D, respectively. The threonine (T) residue in the AMPK motif that is highlighted in yellow color was mutated alanine (A) for *pmitoAIP(TA)* for Fig. 3A-B); **B**. Experimental design for co-transfection studies presented in Fig. 3C-D; and **C**. C2C12 myoblasts were transfected with *pmitoAIP* or the control plasmid *pmitoAIP(TA)* for 24 hours followed by treatment with oligomycin (10 μM) and antimycin A (4 μM) (OA) for 3 hours and stained with MitoTracker^{TM} (0.5 μM) (Green) for 30 min and DAPI (3.575 μM) (Blue) for 10 min before fixation with 4% paraformaldehyde for epifluorescence microscopy. The inserts show enlarged image of the indicated area of the mitochondrial network. Scale bar = 10 μm; D. Quantification of mitochondrial network complexity (measured as 1-cell_mean_mito_euler_number) by MitoHacker analysis. *** denotes $p < 0.001$.

Table S1. List of native and associated mitochondrial proteins found in protein-protein interaction bioinformatic results for AMPKα1.

AMPK alpha 1							
**Protein Name**	**Protein Abbreviation**	**Gene**	**Organism**	**Location**	**Function**	**Key**	**Database**
Voltage-dependent anion-selective channel protein 3	Vdac3	Vdac3	Rattus norvegicus (Rat)	OMM	Diffusion of small hydrophillic molecules	Membrane Potential	IntAct
Voltage-dependent anion-selective channel protein 1	VDAC1	VDAC1	Homo sapiens (Human)	OMM	Diffusion of small hydrophillic molecules	Membrane Potential	IntAct
Voltage-dependent anion-selective channel protein 2	Vdac2	Vdac2	Rattus norvegicus (Rat)	OMM	Diffusion of small hydrophillic molecules	Membrane Potential	IntAct
Voltage-dependent anion-selective channel protein 1	Vdac1	Vdac1	Rattus norvegicus (Rat)	OMM	Diffusion of small hydrophillic molecules	Membrane Potential	IntAct
ATP synthase subunit beta, mitochondrial	F1Atp-ase β	Atp5f1b	Rattus norvegicus (Rat)	IMM	Forms part of the extramembraneous catalytic core of ATP synthase that produces ATP from ADP	ATP Production	Biogrid / IntAct
ATP synthase subunit beta, mitochondrial	F1ATP-ase β	ATP5F1B	Homo sapiens (Human)	IMM	Forms part of the extramembraneous catalytic core of ATP synthase that produces ATP from ADP	ATP Production	IntAct
ATP synthase subunit alpha, mitochondrial	f1Atp-ase α	Atp5f1a	Rattus norvegicus (Rat)	IMM	Forms part of the extramembraneous catalytic core of ATP synthase that produces ATP from ADP	ATP Production	Biogrid / IntAct
ATP synthase subunit gamma, mitochondrial	f1Atp-ase γ	Atp5f1c	Rattus norvegicus (Rat)	IMM	Forms part of the extramembraneous catalytic core of ATP synthase that produces ATP from ADP	ATP Production	Biogrid / IntAct
ATP synthase subunit O, mitochondrial	Atp5O	Atp5po	Rattus norvegicus (Rat)	IMM	Forms part of the extramembraneous catalytic core of ATP synthase that produces ATP from ADP	ATP Production	Biogrid / IntAct
Mitochondrial dicarboxylate carrier	Dic	Slc25a10	Rattus norvegicus (Rat)	IMM	Forms part of the extramembraneous catalytic core of ATP synthase that produces ATP from ADP	ATP Production	Biogrid / IntAct
Phosphate carrier protein, mitochondrial	Ptp	Slc25a3	Rattus norvegicus (Rat)	IMM	ADP with mitochondrial ATP across the IMM	ATP Production	Biogrid / IntAct
Tricarboxylate transport protein, mitochondrial	Ctp	Slc25a1	Rattus norvegicus (Rat)	IMM	ADP with mitochondrial ATP across the IMM	ATP Production	IntAct
Mitochondrial 2-oxoglutarate/malate carrier protein	Ogcp	Slc25a11	Rattus norvegicus (Rat)	IMM	ADP with mitochondrial ATP across the IMM	ATP Production	IntAct
ADP/ATP translocase 1	Ant1	Slc25a4	Rattus norvegicus (Rat)	IMM	ADP with mitochondrial ATP across the IMM	ATP Production	Biogrid / IntAct
ADP/ATP translocase 2	Ant2	Slc25a5	Rattus norvegicus (Rat)	IMM	ADP with mitochondrial ATP across the IMM	ATP Production	Biogrid
ADP/ATP translocase 2	ANT2	SLC25A5	Homo sapiens (Human)	IMM	ADP with mitochondrial ATP across the IMM	ATP Production	IntAct
2-oxoisovalerate dehydrogenase subunit beta, mitochondrial	Bckdhβ	Bckdhb	Rattus norvegicus (Rat)	Matrix	Catalyzes the conversion of alpha-keto acids to acyl-CoA and CO2	ATP Production	IntAct
2-oxoisovalerate dehydrogenase subunit alpha, mitochondrial	Bckdhα	Bckdha	Rattus norvegicus (Rat)	Matrix	Catalyzes the conversion of alpha-keto acids to acyl-CoA and CO2	ATP Production	IntAct
Dihydrolipoamide acetyltransferase component of pyruvate dehydrogenase complex	Bckd	Dbt	Rattus norvegicus (Rat)	Matrix	Catalyzes the conversion of alpha-keto acids to acyl-CoA and CO2	ATP Production	IntAct
Isocitrate dehydrogenase [NADP], mitochondrial	Idh	Idh2	Rattus norvegicus (Rat)	Matrix	Intermediary metabolism and energy production - may associate/interact with pyruate dehydrogenase	ATP Production	IntAct
Argininosuccinate Synthase	AS	Ass1	Rattus norvegicus (Rat)	Multiple (including Mitochondria)	urea cycle	ATP Production	IntAct
Pyruvate carboxylase, mitochondrial	PCB	PC	Homo sapiens (Human)	Matrix	and transfer of carboxyl groups to pyruvate	ATP Production	IntAct
3-ketoacyl-CoA thiolase, mitochondrial	Thiolase 1	Acaa2	Rattus norvegicus (Rat)	Matrix	Fatty acid metabolism and Bnip3 regulation	ATP Production / Proteostasis	IntAct
NFU1 iron-sulfur cluster scaffold homolog, mitochondrial	NFU1	NFU1	Homo sapiens (Human)	IMM/Cytosol	Fe-S cluster scaffold protein	ATP Production / Proteostasis	IntAct
Prohibitin-2	PHB2	PHB2	Homo sapiens (Human)	Nucleus/Mito	regulates cytochrome-c oxidaase assembly and respiration	ATP Production / Proteostasis	IntAct
Prohibitin	Phb	Phb	Rattus norvegicus (Rat)	IMM	inhibits DNA synthesis, regulates mito respiration, aging	ATP Production / Proteostasis	IntAct
Prohibitin	PHB	PHB	Homo sapiens (Human)	IMM	inhibits DNA synthesis, regulates mito respiration, aging	ATP Production / Proteostasis	IntAct
Poly [ADP-ribose] polymerase 1	PARP-1	PARP1	Homo sapiens (Human)	Nucleus / Mitochondria	NAD binding and mitochondria organization	ATP Production / Proteostasis	IntAct
DnaJ homolog subfamily A member 1	DNAJA1	DNAJA1	Homo sapiens (Human)	Nucleus / ER / Mitochondria	mitochondria protein import / co-chaperone for HSPA1B / inhibits translocation of BAX to mitochondria	ATP Production / Proteostasis	IntAct
DnaJ homolog subfamily A member 1	Dnaja1	Dnaja1	Rattus norvegicus (Rat)	Nucleus / ER / Mitochondria	chaperone for HSPA1B / inhibits translocation of BAX to mitochondria	ATP Production / Proteostasis	IntAct

Table S1 (continued)

AMPK alpha 1 cont.							
**Protein Name**	**Protein Abbreviation**	**Gene**	**Organism**	**Location**	**Function**	**Key**	**Database**
Cytochrome b-c1 complex subunit 2, mitochondrial	Uqcrc2	Uqcrc2	Rattus norvegicus (Rat)	IMM	Required for assembly of ubiquinol-cytochrome c reductase complex	Proteostasis	IntAct
60 kDa heat shock protein, mitochondrial	mtHsp60	Hspd1	Rattus norvegicus (Rat)	Matrix	Mitochondrial protein import and folding	Proteostasis	IntAct
Mitochonrial import receptor subunit TOM34	Tom34	Tomm34	Rattus norvegicus (Rat)	OMM	Import of cytosolic synthesized proteins into mitochondria	Proteostasis	Gene Mania
Mitochonrial import receptor subunit TOM34	TOM34	TOMM34	Homo sapiens (Human)	OMM	Import of cytosolic synthesized proteins into mitochondria	Proteostasis	Biogrid
mitochondrial translation initiation factor IF-2	IF-2Mt	MTIF2	Homo sapiens (Human)	Matrix	Mitochondrial protein synthesis, binding of tRNA to 30S ribosomal subunits, and GTP hydrolysis	Proteostasis	Biogrid
probable leucine-tRNA ligase	LeuRS	LARS2	Homo sapiens (Human)	Matrix	Mitochondrial protein synthesis, Aminoacyl-tRNA synthetase	Proteostasis	Biogrid
Mitochondrial fission regulator 2	Mtfr2	Mtfr2	Mus musculus (Mouse)	n/a	Mitochondrial fission	Proteostasis	Biogrid / Gene Mania / IntAct
Ganglioside-induced differentiation-associated protein 1	GDAP1	GDAP1	Homo sapiens (Human)	OMM	mitochondrial fission (PMID: 16172208)	Proteostasis	IntAct
MICOS complex subunit Mic60	Immt	Immt	Rattus norvegicus (Rat)	IMM	maintains crista junctions, IMM architecture, formation of contact sites to the OMM	Proteostasis	IntAct
Heat shock 70 kDa protein 1B	Hsp70-1b	Hspa1b	Rattus norvegicus (Rat)	Multiple (including Mitochondria)	protein folding and chaperone	Proteostasis	IntAct
Heat shock protein 90-beta	HSP90β	HSP90AB1	Homo sapiens (Human)	Multiple (including Mitochondria)	protein folding and chaperone	Proteostasis	IntAct
Hetergeneous nuclear ribonucleoprotein K	hnRnpK	Hnrnpk	Rattus norvegicus (Rat)	Multiple (including Mitochondria)	mRNA processing	Proteostasis	IntAct
Serine/threonine-protein kinase Ulk1	Ulk1	Ulk1	Mus musculus (Mouse)	Multiple (including Mitochondria)	mitophagy initiation	Proteostasis	IntAct
Serine/threonine-protein kinase Ulk1	ULK1	ULK1	Homo sapiens (Human)	Multiple (including Mitochondria)	mitophagy initiation	Proteostasis	IntAct
Progranulin	Progranulin	Grn	Mus musculus (Mouse)	Multiple (including Mitochondria)	Lysome regulation	Proteostasis	IntAct
Glycine--tRNA ligase	Glycine--tRNA ligase	Gars2	Rattus norvegicus (Rat)	Mitocondria / Exosomes	catalyzes ligation of glycine at 3'-end of tRNA	Proteostasis	IntAct
Trafficking kinesin-binding protein 2	TRAK2	TRAK2	Homo sapiens (Human)	Mitochondria / Endosomes	Lysome trafficking	Proteostasis	IntAct
Endoplasmic reticulum chaperone BiP	BiP	Hspa5	Rattus norvegicus (Rat)	Multiple (including Mitochondria)	stablizes mitochondrial membrane	Proteostasis	IntAct
Endoplasmic reticulum chaperone BiP	BiP	HSPA5	Homo sapiens (Human)	Multiple (including Mitochondria)	stablizes mitochondrial membrane	Proteostasis	IntAct
Elongation factor Tu, mitochondrial	Ef-Tu	Tufm	Rattus norvegicus (Rat)	n/a	Recruitment of Atg5-Atg12 and Nlrx1 for autophagy, inhibition of Rlr-mediated type I interferon	Proteostasis	IntAct
40S ribosomal protein S3	RPS3	RPS3	Homo sapiens (Human)	IMM / Nucleus / Cytoskeleton	reduces cellular ROS levels and mito DNA damage	Redox	IntAct
40S ribosomal protein S3	Rps3	Rps3	Rattus norvegicus (Rat)	IMM / Nucleus / Cytoskeleton	reduces cellular ROS levels and mito DNA damage	Redox	IntAct
Peroxiredoxin-1	Prdx1	Prdx1	Rattus norvegicus (Rat)	Matrix / Cytosol / Nucleus / Peroxisomes	Catlyzes reduction of hydrogen peroxide to water	Redox	IntAct
Myotonin-protein kinase	Dmpk	Dmpk	Mus musculus (Mouse)	OMM / Nucleus / ER / Cytosol	contraction conduction and calcium homeostasis	Signaling	Gene Mania
Induced myeloid leukemia cell differentiation protein Mcl-1	BCL-2-L-3	MCL	Homo sapiens (Human)	Nucleus / Cytosol/ Mitochondria	link between growth factor and apoptotic pathways	Signaling	IntAct
Bcl2-associated agonist of cell death	BCL2	BAD	Homo sapiens (Human)	Cytosol / OMM	link between growth factor and apoptotic pathways	Signaling	IntAct
Cell death activator CIDE-A	Cidea	Cidea	Mus musculus (Mouse)	Multiple (including Mitochondria)	Lipid storage and apoptosis	Signaling	IntAct
A-kinase anchor protein SPHKAP	Akap-S	Sphkap	Rattus norvegicus (Rat)	Cytosol / Mitochondria	cAMP type I regulatory subunit binding anchor	Signaling	IntAct
Receptor of activated protein C kinase 1	RACK1	Rack1	Homo sapiens (Human)	Multiple (including Mitochondria)	Scaffold protein for a variety of signaling proteins	Signaling	IntAct
Receptor of activated protein C kinase 1	Rack1	Rack1	Mus musculus (Mouse)	Multiple (including Mitochondria)	Scaffold protein for a variety of signaling proteins	Signaling	IntAct
Tyrosine-protein kinase Fyn	FYN	FYN	Homo sapiens (Human)	Multiple (including Mitochondria)	NOX4 regulation	Signaling	IntAct
Serine/threonine-protein kinase STK11	LKB1	STK11	Homo sapiens (Human)	Mitocondria / Cytosol	AMPK agonist, phosphorylates T-loop of AMPKa1 & a2	Signaling	Biogrid

Table S2. List of native and associated mitochondrial proteins found in protein-protein interaction bioinformatic results for AMPKα2.

AMPK alpha 2							
**Protein Name**	**Protein Abbreviation**	**Gene**	**Organism**	**Location**	**Function**	**Key**	**Database**
Probable proline--tRNA ligase, mitochondrial	ProRS	PARS2	Homo sapiens (Human)	Matrix	prolyl-tRNA synthetase	ATP Production / Proteostasis	Biogrid / IntAct
Protein NipSnap homolog 1	NIPSNAP1	NIPSNAP1	Homo sapiens (Human)	IMM space / Plasma Membrane	neurotransmitter binding	Proteostasis	IntAct
Iron-sulfur cluster co-chaperone protein HscB	C-HSC20	HSCB	Homo sapiens (Human)	Mitochondria / Cytoplasm	co-chaperone in iron-sulfur cluster assembly	Proteostasis	IntAct
Pleckstrin homology domain-containing family N member 1	CKIP-1	PLEKHN1	Homo sapiens (Human)	Mitochondria / Plasma Membrane	mRNA stability, cardiolipin binding, promotes apoptosis by enhancing BAX-BAK	Proteostasis	IntAct
Dynamin-2	Dynamin-2	DNM2	Homo sapiens (Human)	Multiple (including Mitochondria)	trafficing	Proteostasis	IntAct
Serine/threonine-protein kinase Ulk1	Ulk1	Ulk1	Mus musculus (Mouse)	Multiple (including Mitochondria)	mitophagy	Proteostasis	IntAct
Serine/threonine-protein kinase Ulk1	ULK1	ULK1	Homo sapiens (Human)	Multiple (including Mitochondria)	mitophagy	Proteostasis	IntAct
Heat shock protein 90-beta	HSP90β	HSP90AB1	Homo sapiens (Human)	Multiple (including Mitochondria)	protein folding and chaperone	Proteostasis	IntAct
Ras-related protein Rab-3D	Rab3d	Rab3d	Mus musculus (Mouse)	Multiple (including Mitochondria)	Protein transport	Proteostasis	IntAct
Pyruvate kinase PKM	PKM	PKM	Homo sapiens (Human)	Multiple (including Mitochondria)	ROS adaptation and inhibition of apoptosis	Proteostasis	IntAct
Peroxisome proliferator-activated receptor gamma coactivator 1-alpha	PGC1α	PPARGC1A	Homo sapiens (Human)	Multiple (including Mitochondria)	Promotes mitochondrial mRNA transcription	Proteostasis	IntAct
Superoxid dismutase	MnSod	Sod2	Rattus norvegicus (Rat)	Matrix	scavager of superoxide anion radical	Redox	Gene Mania
Serine/threonine-protein kinase STK11	Lkb1	Stk11	Rattus norvegicus (Rat)	Mitocondria / Cytosol	AMPK agonist, phosphorylates T-loop of AMPKa1 & a2	Signaling	Biogrid
Serine/threonine-protein kinase STK11	LKB1	STK11	Homo sapiens (Human)	Mitocondria / Cytosol	AMPK agonist, phosphorylates T-loop of AMPKa1 & a2	Signaling	Biogrid / Gene Mania / IntAct

Table S3. Significance table for quantification of mitochondrial morphological parameters with comparisons among untransfected and mitoAIP- and mitoAIP(TA)-transfected C2C12 myoblasts treated with DMSO or OA. Red color highlights $p < 0.05$.

FEATURE	UNTFX DMSO vs UNTFX OA	UNTFX OA vs mitoAIP OA	UNTFX DMSO vs mitoAIP OA	UNTFX DMSO vs mitoAIP DMSO	UNTFX DMSO vs mitoAIP(TA) DMSO	UNTFXD OA vs mitoAIP(TA) OA	UNTFXD DMSO vs mitoAIP(TA) OA	mitoAIP DMSO vs mitoAIP(TA) DMSO	mitoAIP OA vs mitoAIP(TA) OA
cell_std_mito_equi_diameter_(pixels)	3.09E-11	6.51E-01	4.02E-10	1.17E-01	4.67E-01	5.12E-01	1.39E-08	1.10E-01	3.52E-01
cell_mean_mito_total_density_(pixels)	4.67E-10	7.09E-01	1.12E-09	1.79E-01	4.71E-01	3.93E-01	2.70E-10	1.39E-01	2.65E-01
cell_mean_mito_area_(pixels_squared)	5.71E-10	6.54E-01	1.60E-09	1.81E-01	5.15E-01	5.29E-01	6.20E-10	1.59E-01	3.38E-01
cell_mean_mito_total_branch_length_(pixels)	7.88E-10	5.73E-01	2.59E-09	1.54E-01	5.49E-01	6.05E-01	1.46E-09	1.57E-01	3.59E-01
cell_mean_mito_perimeter_(pixels)	9.70E-10	5.96E-01	3.57E-09	1.78E-01	5.66E-01	8.17E-01	4.22E-09	1.80E-01	5.30E-01
cell_mean_mito_form_factor	2.20E-09	6.34E-01	9.94E-09	1.75E-01	6.31E-01	9.16E-01	5.85E-08	2.06E-01	8.03E-01
cell_mean_mito_branch_count	3.76E-09	7.83E-01	7.26E-09	1.44E-01	4.65E-01	4.21E-01	1.97E-08	1.16E-01	3.41E-01
cell_mean_mito_minor_axis_(pixels)	4.38E-09	9.02E-01	2.43E-08	7.94E-01	5.97E-02	4.89E-01	6.47E-09	5.85E-02	4.54E-01
cell_std_mito_total_branch_length_(pixels)	7.97E-09	6.75E-01	3.76E-08	8.68E-02	8.44E-01	2.68E-01	1.35E-08	2.18E-01	1.86E-01
cell_std_mito_area_(pixels_squared)	8.45E-09	9.13E-01	1.93E-08	9.91E-02	8.16E-01	1.50E-01	5.05E-09	2.20E-01	1.51E-01
cell_std_mito_total_density	9.18E-09	9.72E-01	1.43E-08	9.45E-02	7.60E-01	7.78E-02	1.41E-09	1.83E-01	9.97E-02
cell_std_mito_perimeter_(pixels)	9.77E-09	7.79E-01	3.76E-08	1.08E-01	8.80E-01	3.60E-01	6.63E-08	2.71E-01	2.94E-01
cell_mean_mito_euler_number	1.34E-08	4.13E-01	7.37E-08	9.62E-02	8.26E-01	8.04E-01	2.41E-08	2.36E-01	4.01E-01
cell_std_mito_major_axis_(pixels)	1.41E-08	6.51E-01	6.75E-08	2.94E-01	7.30E-01	6.75E-02	1.31E-08	3.35E-01	2.14E-01
cell_std_mito_minor_axis_(pixels)	1.56E-08	3.84E-01	2.74E-09	6.90E-01	1.04E-01	5.68E-02	5.96E-09	8.67E-02	2.28E-01
cell_std_mito_form_factor	1.86E-08	6.48E-01	1.31E-07	1.23E-01	9.52E-01	7.07E-01	1.41E-06	3.36E-01	5.18E-01
cell_std_mito_branch_count	1.89E-08	7.48E-01	6.71E-08	8.42E-02	7.18E-01	7.77E-02	1.55E-09	1.59E-01	6.06E-02
cell_std_mito_euler_number	6.36E-08	3.17E-01	1.70E-06	3.15E-02	9.98E-01	7.18E-01	3.61E-07	1.63E-01	2.94E-01
cell_mito_count	8.05E-08	5.63E-01	8.53E-04	5.04E-01	4.89E-02	7.94E-01	1.59E-04	3.01E-01	7.34E-01
cell_mean_mito_equi_diameter_(pixels)	1.23E-07	3.36E-01	5.52E-06	2.94E-01	2.15E-01	6.14E-01	2.03E-05	5.69E-02	7.83E-01
cell_std_mito_weighted_cent_x_(pixels)	1.97E-07	9.57E-01	3.70E-07	7.06E-01	7.13E-02	3.51E-02	3.28E-09	1.94E-01	6.38E-02
cell_network_fractal_dimension	2.12E-07	3.83E-01	9.33E-05	8.96E-02	2.07E-01	9.05E-01	1.57E-04	2.43E-02	4.58E-01
cell_std_mito_weighted_distance_(pixels)	2.25E-07	8.92E-01	3.77E-07	7.51E-01	8.89E-02	1.81E-02	1.90E-09	2.16E-01	4.61E-02
cell_std_mito_weighted_cent_y_(pixels)	2.92E-07	8.26E-01	4.40E-07	7.97E-01	1.11E-01	9.24E-03	1.29E-09	2.39E-01	3.48E-02
cell_mean_mito_major_axis_(pixels)	5.16E-07	7.50E-01	6.15E-06	3.35E-01	2.66E-01	7.20E-01	7.22E-06	7.85E-02	5.86E-01
cell_std_mito_average_density_(pixels)	3.42E-06	6.22E-01	8.22E-04	4.59E-01	2.99E-01	7.85E-02	5.82E-02	1.34E-01	2.29E-01
cell_std_mito_solidity	1.04E-04	9.60E-01	3.42E-03	7.10E-01	1.22E-01	7.56E-01	3.31E-03	9.51E-02	7.70E-01
cell_std_mito_median_density_(pixels)	3.53E-04	7.78E-01	1.34E-02	9.51E-01	8.41E-01	1.38E-01	3.92E-01	8.83E-01	2.50E-01
cell_mean_mito_weighted_cent_x_(pixels)	3.88E-04	6.34E-01	1.69E-03	1.80E-01	1.19E-02	1.72E-01	9.19E-05	1.73E-01	1.85E-01
cell_mean_mito_weighted_distance_(pixels)	4.05E-04	6.76E-01	1.71E-03	1.96E-01	1.38E-02	1.01E-01	6.93E-05	1.78E-01	1.56E-01
cell_mean_mito_weighted_cent_y_(pixels)	4.48E-04	7.17E-01	1.82E-03	2.15E-01	1.61E-02	5.72E-02	5.60E-05	1.85E-01	1.32E-01
cell_std_mito_std_branch_length_(degrees)	7.85E-04	6.00E-01	1.00E-02	4.40E-01	2.30E-01	5.72E-02	6.21E-01	1.09E-01	1.23E-01
cell_mean_mito_std_branch_length_(pixels)	9.74E-04	5.17E-01	1.03E-02	9.78E-01	1.21E-01	2.50E-02	1.38E-01	1.28E-01	1.50E-01
cell_std_mito_extent	2.69E-03	1.34E-01	1.52E-01	2.01E-01	4.91E-01	2.14E-01	4.55E-01	1.08E-01	7.56E-01
cell_mean_mito_solidity	4.13E-03	7.38E-01	8.28E-03	7.16E-01	2.65E-01	5.69E-01	1.49E-01	2.03E-01	4.69E-01
cell_mean_mito_distance_(pixels)	2.84E-02	3.52E-01	3.96E-01	7.91E-01	3.56E-01	6.80E-01	2.59E-01	7.75E-01	4.45E-01
cell_std_mito_eccentricity	4.23E-02	1.82E-01	8.87E-03	3.94E-01	1.80E-01	7.93E-01	1.55E-01	4.58E-01	4.90E-01
cell_weighted_kurtosis_y	5.48E-02	7.73E-01	9.83E-02	4.58E-01	2.65E-01	1.53E-01	7.69E-01	9.62E-01	1.44E-01
cell_mean_mito_centroid_y_(pixels)	6.32E-02	3.98E-02	6.96E-01	4.13E-01	3.10E-01	5.81E-01	8.64E-01	9.77E-01	7.28E-01
cell_kurtosis_squared	6.52E-02	8.87E-01	1.44E-01	4.39E-01	2.14E-01	6.33E-01	3.43E-01	8.59E-01	6.22E-01
cell_median_mito_distance_(pixels)	6.97E-02	5.12E-01	3.69E-01	9.38E-01	3.39E-01	4.57E-01	1.62E-01	6.62E-01	3.22E-01
cell_kurtosis_x	7.45E-02	4.59E-01	5.34E-01	7.37E-01	8.04E-01	1.09E-01	8.77E-01	9.40E-01	4.93E-01
cell_kurtosis_y	8.14E-02	6.17E-01	3.37E-01	6.17E-01	7.50E-01	3.78E-01	9.78E-01	8.44E-01	5.84E-01
cell_mean_mito_extent	8.76E-02	5.00E-01	4.79E-02	2.76E-01	4.69E-01	6.65E-01	5.56E-01	1.29E-01	4.53E-01
cell_weighted_skewness_y	1.07E-01	8.08E-01	1.71E-01	4.40E-01	2.71E-01	2.13E-01	6.52E-01	8.49E-01	2.02E-01
cell_mean_mito_std_branch_angle_(degrees)	1.14E-01	9.76E-01	2.05E-01	8.00E-01	3.05E-01	2.36E-01	8.84E-01	2.50E-01	3.12E-01
cell_weighted_kurtosis_x	1.22E-01	6.12E-01	1.12E-01	4.26E-01	4.85E-01	3.02E-01	9.36E-01	7.11E-01	2.09E-01
cell_median_mito_centroid_x_(pixels)	1.24E-01	5.87E-01	7.50E-02	7.25E-01	9.43E-01	3.33E-01	1.15E-01	7.20E-01	4.72E-01
cell_std_mito_roundness	1.28E-01	2.20E-01	8.63E-01	5.35E-02	8.81E-01	3.56E-01	9.87E-01	1.57E-01	9.13E-01
cell_network_effective_extent	1.37E-01	2.39E-01	8.38E-01	2.40E-01	5.31E-01	9.44E-01	2.31E-01	1.58E-01	2.75E-01
cell_std_mito_std_branch_angle_(degrees)	1.42E-01	7.91E-01	3.52E-01	4.47E-01	2.20E-01	2.13E-02	2.38E-01	1.15E-01	5.68E-02
cell_std_mito_mean_branch_length_(pixels)	1.51E-01	5.89E-01	7.26E-01	8.37E-01	3.06E-01	5.42E-01	8.53E-01	3.55E-01	9.12E-01
cell_median_mito_solidity	1.67E-01	5.43E-01	9.57E-02	8.34E-01	2.99E-01	2.37E-01	9.96E-01	5.57E-01	1.41E-01
cell_median_mito_centroid_y_(pixels)	1.80E-01	1.52E-01	9.80E-01	2.11E-01	3.52E-01	6.77E-01	8.44E-01	6.68E-01	8.51E-01

Table S3 (continued)

cell_mean_mito_centroid_x_(pixels)	1.86E-01	4.22E-01	7.68E-02	7.62E-01	7.58E-01	1.59E-01	6.11E-02	6.63E-01	3.06E-01
cell_skewness_y	1.87E-01	1.27E-01	5.77E-01	1.29E-01	8.14E-01	6.85E-01	7.06E-01	1.86E-01	4.60E-01
cell_weighted_skewness_x	1.96E-01	6.51E-01	1.82E-01	4.11E-01	4.00E-01	3.58E-01	8.00E-01	8.53E-01	2.71E-01
cell_median_mito_weighted_cent_x_(pixels)	2.04E-01	3.85E-01	5.77E-01	4.19E-02	5.23E-02	6.80E-01	1.75E-01	8.81E-01	3.21E-01
cell_mean_mito_mean_branch_length_(pixels)	2.06E-01	4.82E-01	7.44E-01	7.72E-01	5.81E-01	7.84E-01	5.97E-01	4.27E-01	8.11E-01
cell_skewness_x	2.12E-01	6.68E-01	1.82E-01	9.52E-01	8.06E-01	2.59E-01	7.60E-01	8.95E-01	1.96E-01
cell_weighted_kurtosis_squared	2.14E-01	8.71E-01	2.37E-01	3.67E-01	6.62E-01	3.24E-02	3.51E-01	4.70E-01	5.39E-02
cell_median_mito_weighted_distance_(pixels)	2.20E-01	4.70E-01	5.38E-01	4.48E-02	4.43E-02	5.57E-01	1.59E-01	9.97E-01	3.21E-01
cell_network_effective_solidity	2.21E-01	1.27E-01	6.26E-01	3.00E-01	6.72E-01	6.64E-01	6.81E-01	5.25E-01	4.45E-01
cell_weighted_skewness_squared	2.26E-01	7.34E-01	2.32E-01	4.31E-01	4.79E-01	1.68E-01	5.20E-01	7.70E-01	1.48E-01
cell_median_mito_weighted_cent_y_(pixels)	2.31E-01	5.46E-01	5.06E-01	3.83E-02	3.82E-02	4.69E-01	1.45E-01	9.93E-01	3.22E-01
cell_mean_mito_average_density_(pixels)	2.48E-01	1.50E-01	8.88E-01	9.30E-01	5.14E-01	4.06E-01	8.22E-01	5.75E-01	7.06E-01
cell_std_mito_mean_branch_angle_(degrees)	2.68E-01	3.21E-01	8.66E-02	9.99E-01	5.18E-01	1.90E-01	8.80E-02	6.17E-01	3.74E-01
cell_median_mito_eccentricity	2.69E-01	2.21E-02	3.27E-01	1.39E-01	9.01E-01	1.63E-01	4.24E-01	2.43E-01	8.48E-01
cell_std_mito_median_branch_length_(pixels)	2.84E-01	6.68E-01	8.06E-01	9.68E-01	3.58E-01	6.89E-01	8.57E-01	4.73E-01	9.76E-01
cell_median_mito_total_density_(pixels)	2.91E-01	3.94E-01	5.32E-01	4.97E-01	2.82E-01	9.72E-01	3.10E-01	5.71E-01	4.80E-01
cell_total_mito_area_(pixels_squared)	2.99E-01	9.92E-01	3.95E-01	5.46E-02	2.70E-01	9.79E-01	3.93E-01	2.36E-01	9.76E-01
cell_median_mito_branch_count	3.21E-01	NA	3.21E-01	3.21E-01	3.21E-01	NA	3.21E-01	NA	NA
cell_median_mito_std_branch_length_(pixels)	3.21E-01	NA	3.21E-01	3.21E-01	3.21E-01	NA	3.21E-01	NA	NA
cell_median_mito_std_branch_angle_(degrees)	3.21E-01	NA	3.21E-01	3.21E-01	3.21E-01	NA	3.21E-01	NA	NA
cell_std_mito_median_branch_angle_(degrees)	3.47E-01	2.45E-01	9.14E-02	9.43E-01	5.50E-01	1.25E-01	6.31E-02	6.65E-01	2.94E-01
cell_median_mito_area_(pixels_squared)	3.50E-01	4.24E-01	6.37E-01	7.73E-01	3.36E-01	9.32E-01	3.65E-01	4.36E-01	4.88E-01
cell_mean_mito_median_branch_length_(pixels)	3.77E-01	4.91E-01	9.59E-01	8.70E-01	6.66E-01	9.58E-01	6.47E-01	5.68E-01	6.93E-01
cell_network_orientation_(degrees)	3.83E-01	7.30E-01	3.71E-01	4.12E-01	4.41E-01	8.86E-01	8.58E-01	9.49E-01	7.59E-01
cell_network_eccentricity	4.10E-01	7.43E-01	3.60E-01	2.96E-01	3.16E-01	5.73E-01	8.97E-01	9.48E-01	4.81E-01
cell_median_mito_total_branch_length_(pixels)	4.14E-01	9.34E-01	4.80E-01	8.97E-01	6.32E-01	7.53E-01	3.72E-01	5.34E-01	7.41E-01
cell_median_mito_major_axis_(pixels)	4.31E-01	9.07E-01	5.12E-01	9.21E-01	6.56E-01	4.50E-01	2.61E-01	7.16E-01	4.91E-01
cell_network_major_axis_(pixels)	4.36E-01	8.61E-01	4.94E-01	1.11E-01	1.68E-01	8.92E-01	6.68E-01	6.98E-01	8.04E-01
cell_mean_mito_median_branch_angle_(degrees)	4.63E-01	2.25E-01	1.12E-01	4.76E-01	8.51E-01	8.43E-01	8.63E-01	6.89E-01	3.89E-01
cell_median_mito_extent	4.72E-01	4.87E-01	2.48E-01	5.36E-01	1.75E-01	9.59E-02	5.15E-02	1.12E-01	2.65E-01
cell_median_mito_perimeter_(pixels)	4.73E-01	5.44E-01	7.76E-01	7.97E-01	5.51E-01	7.00E-01	3.84E-01	4.06E-01	4.29E-01
cell_median_mito_form_factor	5.09E-01	5.80E-01	3.41E-01	8.53E-01	2.14E-01	3.55E-01	2.25E-01	2.42E-01	6.75E-01
cell_mean_mito_orientation_(degrees)	5.62E-01	2.03E-01	1.18E-01	7.31E-01	9.84E-01	7.71E-01	9.96E-01	7.95E-01	3.73E-01
cell_network_minor_axis_(pixels)	5.88E-01	5.69E-01	9.19E-01	2.04E-01	6.33E-01	7.87E-01	9.02E-01	4.33E-01	8.44E-01
cell_mean_mito_roundness	5.90E-01	2.20E-01	1.58E-01	1.91E-01	7.29E-01	3.91E-01	2.78E-01	1.77E-01	8.89E-01
cell_mean_mito_mean_branch_angle_(degrees)	6.21E-01	2.28E-01	1.56E-01	4.75E-01	7.54E-01	9.17E-01	8.93E-01	7.49E-01	4.47E-01
cell_median_mito_equi_diameter_(pixels)	6.49E-01	4.16E-01	8.87E-01	8.60E-01	5.57E-01	9.67E-01	7.23E-01	4.79E-01	5.58E-01
cell_median_mito_mean_branch_length_(pixels)	6.51E-01	8.79E-01	7.94E-01	7.78E-01	5.08E-01	5.96E-01	4.46E-01	4.24E-01	5.79E-01
cell_std_mito_orientation_(degrees)	6.86E-01	2.46E-01	2.11E-01	6.64E-01	9.40E-01	6.47E-02	5.28E-02	7.73E-01	2.75E-01
cell_std_mito_distance_(pixels)	6.87E-01	9.45E-01	8.47E-01	2.36E-02	6.55E-01	6.05E-01	7.78E-01	2.02E-01	7.00E-01
cell_median_mito_average_density_(pixels)	7.17E-01	1.78E-01	4.75E-01	7.55E-01	4.63E-01	9.96E-01	8.12E-01	7.16E-01	3.99E-01
cell_std_mito_centroid_x_(pixels)	7.22E-01	3.97E-01	6.06E-01	1.57E-01	4.87E-02	2.82E-01	5.11E-01	9.74E-01	9.60E-01
cell_median_mito_median_density	7.27E-01	8.37E-01	9.12E-01	6.08E-01	2.39E-01	1.97E-03	2.96E-02	6.14E-01	5.60E-02
cell_median_mito_mean_branch_angle_(degrees)	7.57E-01	9.66E-02	2.28E-01	7.91E-01	5.87E-01	2.43E-01	5.21E-01	4.67E-01	4.70E-01
cell_skewness_squared	8.01E-01	5.98E-01	7.43E-01	6.37E-01	5.23E-01	1.05E-03	1.59E-03	3.73E-01	1.82E-02
cell_mean_mito_eccentricity	8.12E-01	7.00E-02	2.06E-01	8.72E-02	9.61E-01	2.49E-01	3.29E-01	1.69E-01	8.24E-01
cell_median_mito_median_branch_angle_(degrees)	8.53E-01	1.64E-01	3.30E-01	4.52E-01	7.37E-01	6.22E-01	8.68E-01	3.01E-01	3.37E-01
cell_median_mito_roundness	8.64E-01	1.25E-01	2.61E-01	4.42E-01	5.94E-01	3.12E-02	6.39E-02	2.67E-01	3.85E-01
cell_median_mito_median_branch_length_(pixels)	8.93E-01	8.81E-01	8.17E-01	8.18E-01	7.94E-01	8.93E-01	9.74E-01	6.74E-01	8.32E-01
cell_median_mito_minor_axis_(pixels)	9.12E-01	1.94E-01	3.53E-01	6.41E-01	5.03E-01	3.72E-01	4.65E-01	8.87E-01	9.81E-01
cell_median_mito_orientation_(degrees)	9.29E-01	1.25E-01	1.75E-01	8.15E-01	7.46E-01	8.27E-01	8.86E-01	6.57E-01	2.47E-01
cell_std_mito_centroid_y_(pixels)	9.62E-01	3.30E-01	3.80E-01	3.42E-01	8.95E-01	6.91E-01	7.22E-01	3.44E-01	7.58E-01
cell_mean_mito_median_density_(pixels)	9.73E-01	3.96E-01	5.22E-01	5.51E-01	8.16E-01	9.11E-01	9.44E-01	7.10E-01	6.23E-01
cell_median_mito_euler_number	NA	NA	NA	NA	NA	NA	NA	NA	NA
***Note: UNTFX stands for untransfected									

Supplemental Movie 1. Electrical stimulation of cultured adult mouse FDB fibers. Movie clip shows contractions of cultured FDB muscle fibers that were electrically stimulated at 100 Hz stimulation frequency, 300 ms stimulation duration every second, 0.3 ms pulse duration, and 15 V electric potential.
